# Myosin 1E localizes to actin polymerization sites in lamellipodia, affecting actin dynamics and adhesion formation

**DOI:** 10.1242/bio.20135827

**Published:** 2013-10-16

**Authors:** Prabuddha Gupta, Nils C. Gauthier, Yu Cheng-Han, Yuan Zuanning, Bruno Pontes, Malte Ohmstede, René Martin, Hans-Joachim Knölker, Hans-Günther Döbereiner, Mira Krendel, Michael Sheetz

**Affiliations:** 1 Mechanobiology Institute, National University of Singapore, Singapore 117411; 2 Institut für Biophysik, Universität Bremen, 28359 Bremen, Germany; 3 Department Chemie, TU Dresden, 01062 Dresden, Germany; 4 Department of Cell and Developmental Biology, SUNY Upstate Medical University, Syracuse, NY 13210, USA; * Present address: Department of Biological Chemistry, IACS, Kolkata 700 032, India

**Keywords:** Myosin 1E, Cell–matrix adhesion, Transport

## Abstract

Because the actin network in active lamellipodia is continuously assembling at the edge, moving inward and disassembling, there is a question as to how actin-binding proteins and other components are transported to the leading edge and how nascent adhesions are stabilized. Active transport could play a significant role in these functions but the components involved are unknown. We show here that Myosin 1E (a long tailed Myosin 1 isoform) rapidly moves to the tips of active lamellipodia and to actin-rich early adhesions, unlike Myosin 1G, 1B or 1C (short tailed isoforms). Myosin 1E co-localizes with CARMIL, FHOD1, Arp3 and β3-integrin in those early adhesions. But these structures precede stable paxillin-rich adhesions. Myosin 1E movement depends upon actin-binding domains and the presence of an SH3 oligomerization domain. Overexpression of a Myosin 1E deletion mutant without the extreme C-terminal interacting (SH3) domain (Myosin 1EΔSH3) increases edge fluctuations and decreases stable adhesion lifetimes. In contrast, overexpression of Myosin 1E full tail domain (TH1+TH2+TH3/SH3) decreases edge fluctuation. In Myosin 1E knockdown cells, and more prominently in cells treated with Myosin 1 inhibitor, cell–matrix adhesions are also short-lived and fail to mature. We suggest that, by moving to actin polymerization sites and early adhesion sites in active lamellipodia, Myosin 1E might play important roles in transporting not only important polymerizing proteins but also proteins involved in adhesion stabilization.

## Introduction

**Fig. 1.**
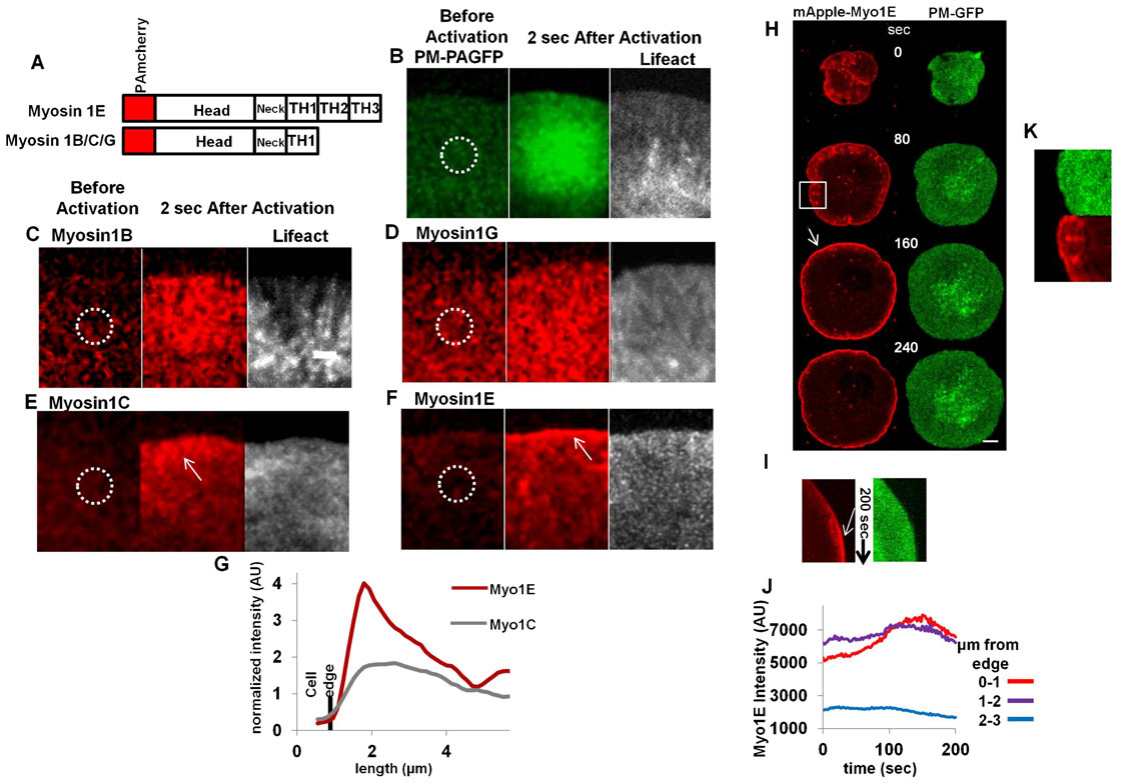
Myosin 1E localizes to tip of spreading lamellipodia. (A) Domain organization of Myosin 1 isoform. (B–F) Photoactivation by 405 nm laser in the ROA (white circle) and observation by 561 nm laser after 2 sec, of (B) Plasma membrane-PAGFP membrane diffusion control, (C) PAmcherry-Myosin 1B, (D) 1G, (E) 1C, (F) 1E. Bar 2 µm, B–F is in same scale. (G) Normalized (against the intensity at ROA) average intensity perpendicular to the long axis of the 2 sec figure panels in Myosin 1C and Myosin 1E. (H) Profile and (I) kymograph of mApple-Myosin 1E and plasma membrane GFP (PM-GFP) in spreading cell. Arrows indicate Myosin 1E accumulation. (J) Quantitation of absolute intensity in Myosin 1E channel in (H) (supplementary material Movie 1). Bar 5 µm. (K) Enlarged view of the boxed region in (H), showing spots in Myosin channel behind the lamellipodial tip.

Myosin 1 proteins are abundant actin-activated ATPases that are commonly monomeric (single headed) when purified (Pollard and Korn, 1973). Another feature of these Myosin 1 isoforms is their association with cell membrane by the Tail Homology 1 (TH1) domain, that contains a lipid-binding, PH like, domain (Hokanson et al., 2006; Patino-Lopez et al., 2010). Myosin 1s are further classified as short tailed (e.g. 1B/1C/1G) or long tailed (e.g. 1E/1F) based on absence or presence of glycine/proline/alanine rich (TH2) and SH3 domains (TH3) in the tail region (Fig. 1A) (McConnell and Tyska, 2010). Myosin 1 isoforms accumulate at the leading edge during cell-spreading or in migrating cells (Fan et al., 2012; Fukui et al., 1989). They are linked to a number of aspects of cell motility including membrane–cytoskeleton adhesion and transport of actin towards the leading edge during cell migration (Fan et al., 2012; McConnell and Tyska, 2010). Specialized actin rich structures like lamellipodia and filopodia are necessary components of migrating cells (Ridley et al., 2003). These structures not only need G-actin flow for actin polymerization at the tip but also need proteins responsible for capping the barbed end of actin filaments and stabilization of newly formed matrix adhesions (Borisy and Svitkina, 2000; Mogilner and Keren, 2009; Schmidt et al., 1993; Small et al., 2002). Myosin 1E is a barbed end directed motor with a tail that contains actin binding (TH2) and protein–protein interaction (SH3) domains (Bement et al., 1994b; Krendel et al., 2007). Also, it is the only such long-tailed Myosin 1 that is ubiquitously expressed (Bement et al., 1994a; Kim et al., 2006). Therefore, Myosin 1E could be a major transporter of proteins involved in actin polymerization and adhesion stabilization in dynamic lamellipodia. Here, we show, Myosin 1E dynamically localizes at the very tip of active actin rich lamellipodia and in stationary actin rich puncta that transiently appear behind the lamellipodial tip, containing FHOD1, Arp3, CARMIL, and β3-integrin. These early structures turn over before nascent adhesions form (Choi et al., 2008). The short-tailed Myosin 1 isoforms (1B/1C/1G) do not co-localize at lamellipodia tips or in puncta. Because expression of Myosin 1E mutants and knockdown of Myosin 1E decreases the maturation of cell–matrix adhesions, we suggest that Myosin 1E moves to actin polymerization sites to support further polymerization and to stabilize matrix adhesions in lamellipodia.

## Results

### Myosin 1E uniquely localizes to lamellipodial tips and spots behind

To determine which Myosin 1s were transported in active lamellipodia, we developed photo-activatable PAmCherry tagged long-tailed Myosin 1E and PAmCherry tagged short-tailed Myosin 1B, 1C, 1G proteins (Fig. 1A). Since the lamellipodium is only ∼200 nm thick (Abercrombie et al., 1971), we controlled for soluble proteins with PA-GFP and for non-specific binding to the plasma membrane with PA-GFP-PM. After transfection in mouse embryonic RPTPα^+/+^ fibroblast cells (Su et al., 1999), photo-activation of PA-GFP or PAmCherry provided high signal to noise (Lukyanov et al., 2005; Subach et al., 2009). Using confocal microscopy and sections that cover the entire lamellipodial thickness, we activated fluorescence in localized regions of activation (ROA) at the back of lamellipodia and observed distribution of fluorescence after 2 sec (Fig. 1B–F). Upon photoactivation, membrane-anchored PM-PAGFP spread uniformly from the ROA in lamellipodia (Fig. 1B). Myosin 1B and Myosin 1G were distributed evenly in the entire lamellipodia (Fig. 1C,D), indicating a similar diffusive movement aided by membrane-bound PH-like domains (Komaba and Coluccio, 2010). Soluble free PA-GFP got spread in both lamellipodia and cell-body (supplementary material Fig. S1A). However, in the cases of Myosin 1C (Fig. 1E) and Myosin 1E (Fig. 1F), the distribution was more biased towards the tips of lamellipodia (white arrows). Myosin 1C was linked to G-actin transport (Fan et al., 2012) and its concentration in 1–2 µm regions at the tips of lamellipodia overlapped with active polymerization zones of actin (Eiseler et al., 2010). We saw that Myosin 1E was concentrated in a narrower band than Myosin 1C, at the tips of lamellipodia (Fig. 1E,F white arrow, Fig. 1G). These experiments were repeated at least 10 times (supplementary material Fig. S2A–C) and showed a dramatic concentration of PAmcherry-Myo1E two sec after photo-activation (Fig. 1G). To get further insight into the tip accumulation, we transfected cells with mApple-Myosin1E and followed the distribution during spreading on fibronectin-coated glass surfaces (supplementary material Movie 1; Fig. 1H,K). Tip accumulation of Myosin 1E increased as the lamellipodia moved forward in the contractile spreading phase and peaked at the end of active spreading (supplementary material Movie 1; Fig. 1H,I white arrows). The increase in accumulation of Myosin 1E at the tips of lamellipodia (Fig. 1J, absolute intensity plot, corresponding to the Kymograph) dissipated when lamellipodia subsequently retracted. This increase was not due to a membrane geometry artifact, since co-transfected Plasma-Membrane bound GFP (Teruel et al., 1999) did not show any such increase over time (Fig. 1H,I; supplementary material Movie 1). Thus, the accumulation of Myosin 1E at the edges of active lamellipodia correlated with the production of an edge extension complex (Bretschneider et al., 2009). Another type of Myosin 1E accumulations were the dot like structures behind the lamellipodial tips that were absent in PM-GFP (Fig. 1K, corresponding to the boxed region of Fig. 1H; supplementary material Movie 1). We further investigated presence of actin and adhesion/polymerization proteins in those spots.

### Myosin 1E localizes to actin-polymerization sites in lamellipodia

**Fig. 2.**
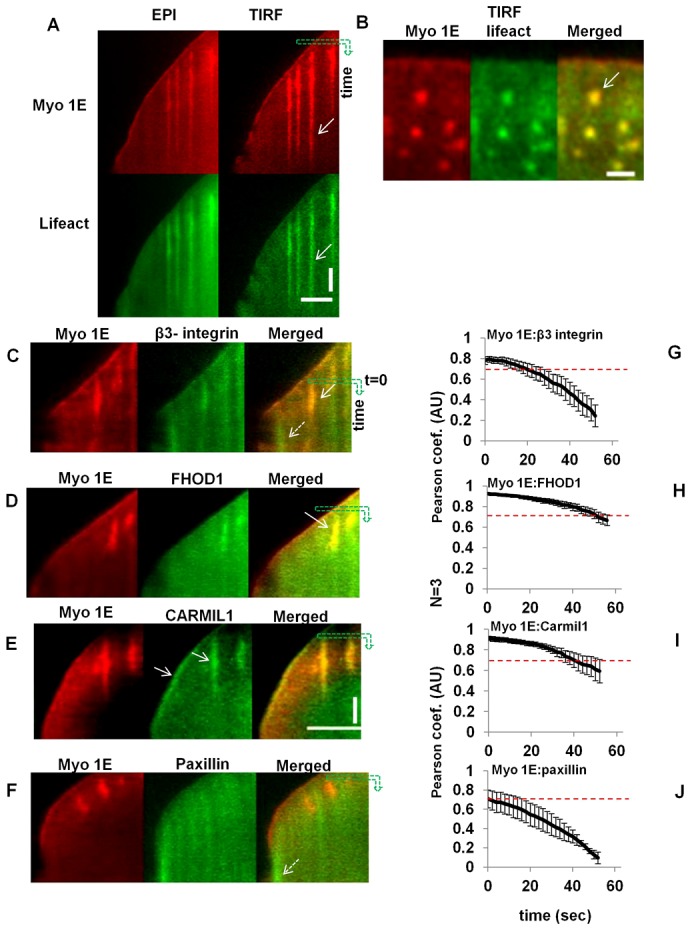
β3-integrin, FHOD and CARMIL co-localize with Myosin 1E in actin rich early adhesion. Paxillin shows partial overlap. (A) Simultaneous observation (kymograph) of Myosin 1E and actin (lifeact) in EPI and TIRF channels (supplementary material Movie 2). Time is along vertical axis along the green arrow, length is along horizontal axis. Bar 5 µm. (B) Co-localization of Myosin 1E with actin rich spots (white arrow). Bar 2 µm. Comparative co-localization of (C) EGFP-β3-integrin, (D) FHOD1, (E) CARMIL1a and (F) Paxillin with mApple-Myosin 1E at the tip and early adhesion structures in spreading lamellipodia (by kymograph, time as vertical and length as horizontal axis). Bar 5 µm, C–F is in same scale, time bar 30 sec. (G–J) Quantification of such overlap of Myosin 1E with β3-integrin/FHOD1/CARMIL1/paxillin by Pearson coefficients (starting point or zero sec is indicated in the kymograph as the starting point of downward green arrows in C–F) (associated supplementary material Movies 3–6).

Several studies have shown that the upper layer lamellipodial actin was distinct from the lower layer and was more rapidly transported inward (Gardel et al., 2010; Giannone et al., 2007; Ponti et al., 2004). Dorsal actin filaments were aligned with the barbed ends roughly pointing to the edge and would support the movement of the Myosin 1 isoforms to the edge (Pollard and Borisy, 2003). In order to determine the relative distribution of actin and Myosin 1E, cells were transfected with EGFP-lifeAct (Fig. 2A bottom) along with mApple-Myosin 1E (Fig. 2A top) and observed by both epifluorescence and TIRF microscopy simultaneously. We observed periodic appearance and disappearance of Myosin 1E in TIRF at the leading edge (Fig. 2A right; supplementary material Movie 2), correlating with similar periodic behavior of f-actin (lifeact) in the TIRF layer (Giannone et al., 2004; Giannone et al., 2007) (Fig. 2A right; supplementary material Movie 2). In epifluorescence, Myosin 1E showed a gradual accumulation at the tip of lamellipodia over time, crowning the sites of actin polymerization (Fig. 2A left). The overall actin distribution was more similar to the Myosin 1C distribution than Myosin 1E, in agreement with role of Myosin 1C in g-actin transport for polymerization in lamellipodia (Fan et al., 2012). Long tailed Myosin 1s, like Myosin 1E, were proposed as carriers of CARMIL to the lamellipodial tip (Jung et al., 2001). CARMIL1, the negative regulator of actin capping protein, has identical localization pattern at the tip of lamellipodia (Yang et al., 2005).

Recent findings indicated that actin polymerized at integrin clusters back from the leading edge in lamellipodia (Galbraith et al., 2002; Ghassemi et al., 2012) and that could have corresponded with the dot-like concentrations of Myosin1E in a similar region. In the TIRF microscope as the lamellipodia spread forward (Fig. 2A right panels), we found <1 µm diameter actin spots where Myosin 1E was concentrated (Fig. 2A,B; supplementary material Fig. S1B–D white arrows; supplementary material Movie 2). Short tailed Myosin 1G had no co-localization with these actin spots (supplementary material Fig. S1E). Myosin 1C was involved in active g-actin transport (Fan et al., 2012), but was also not found in this type of punctuate structures (Bose et al., 2004; Hokanson et al., 2006). These spots had greater contrast in TIRF than in epifluorescence of both channels (Fig. 2A). Because these spots were stationary, actin rich, and in the TIRF field, they appeared similar to sites of actin polymerization at integrin clusters observed previously (Yu et al., 2011). These stationary spots were fibronectin substrate dependent. In cells spread on a poly-L-Lys substrate, similar spots were more dynamic and had comet like structures (Ryan et al., 2012). They were not linked to clathrin mediated endocytic pathways as these actin rich puncta temporally preceded clathrin rich spots on ventral surfaces of lamellipodia (supplementary material Fig. S3A,B) (Cheng et al., 2012).

To better understand the origin of the spots that were back from the leading edge, we co-transfected mApple-Myosin 1E with several components of early adhesions at sites of integrin clustering. Because of its involvement in early adhesions, we initially co-transfected GFP-β3-integrin (supplementary material Movie 3) with mApple-Myosin 1E. There was an overlap between Myosin 1E and β3-integrin at the tip of lamellipodia and at the spots behind tip of lamellipodia (Fig. 2C white solid arrow; supplementary material Movie 3; Fig. S4A light blue circles). Although Myosin 1E and β3-integrin were aligned in spots, their intensities did not vary in unison, especially during the contractile phase of spreading (P2) (Fardin et al., 2010) (supplementary material Movie 3; Fig. 2C white dotted arrow), when, Myosin 1E intensity gradually deceased, but a constant staining of β3-integrin was observed in patches. These differences might be due to longer lived adhesion formation containing β3-integrin (supplementary material Movie 3; Fig. 2C white dotted arrow).

Formins were implicated in actin polymerization at these integrin clusters (Yu et al., 2011; Thomas Iskratsch and colleagues, personal communication); therefore, we next co-transfected GFP-FHOD1 with mApple-Myosin 1E (supplementary material Movie 4). There was co-localization of Myosin 1E and FHOD1 at spots in motile lamellipodia (Fig. 2D white arrow; supplementary material Movie 4; Fig. S4B dark blue circle). However, FHOD1 was not found at lamellipodial tips and was primarily cytosolic (supplementary material Movie 4; Fig. 2D). The intensities of FHOD1 and Myosin 1E were found to change in unison during the protrusive and contractile phases of lamellipodia spreading (supplementary material Movie 4). Logically, the cargo of Myosin 1E, CARMIL (Jung et al., 2001; Liang et al., 2009; Yang et al., 2005) should also have been concentrated at the spots if Myosin 1E was actively transporting material to the spots. Indeed, CARMIL and Myosin 1E were co-localized in the spots, as well as at the tip of lamellipodia (Fig. 2A,E arrows; supplementary material Fig. S4C dark blue circle; supplementary material Movie 5). Changes of fluorescence intensities in both channels were also correlated (supplementary material Movie 5). Thus, there was a strong correlation in time and space between the actin-polymerizing protein FHOD1 and Myosin 1E concentration at the spots as well as CARMIL, the logical cargo of Myosin 1E.

If the spots were at sites of integrin concentration, then there may have been other integrin-associated adhesion proteins at these sites. To test this hypothesis, GFP-paxillin was co-transfected with mApple-Myosin 1E. As reported previously (Choi et al., 2008), GFP-paxillin was not seen initially at the tip (Fig. 2F initial spreading; supplementary material Fig. S4D; Movie 6), but appeared strongly with the onset of contractile spreading phase (Fig. 2F white dotted arrow; supplementary material Movie 6). Further, changes in paxillin intensity at the lamellipodial tip did not change in unison with that of Myosin 1E (supplementary material Movie 6; Fig. 2F). As a spreading lamellipodium moved forward, stopped and constricted back, Myosin 1E intensity at lamellipodial edge increased, reaching a maximum and decreased during the periodic contractile cycle. On the other hand, paxillin maintained a constant intensity, behind the lamellipodia tip until the contractile phase and then it started to mature behind Myosin 1E. Paxillin was present in nascent adhesions behind the tip but these only overlapped partially with the spots of Myosin 1E (Fig. 2F; supplementary material Movie 6). As paxillin intensity stabilized, Myosin 1E intensity went down (supplementary material Movie 6; Fig. 2F). All spots of Myosin 1E turned over before maturation of paxillin at the edge (supplementary material Movie 6; Fig. 2F).

To quantify the level of co-localization of the various components in the spots, we calculated the variance of Pearson coefficient between Myosin 1E and other proteins in lamellipodial region for one minute of spreading (Fig. 2G–J). Since the cytosolic fluorescence intensity levels of these proteins were high, all gave Manders overlap ∼90% all places (data not shown). We focused on whether the Pearson coefficient stayed above 0.7, which indicated that the intensity variations of both channels were in unison. For Paxillin, the initial Pearson coefficient indicated overlap (0.7) (Fig. 2J; supplementary material Movie 6), but soon fell below that value. For FHOD1 and CARMIL, the Pearson coefficient of overlap was >0.7 during the entire course of movie, supporting the observation that these proteins were bound in the same regions (Fig. 2H–I). For β3-integrin, initially there was good overlap, but within 20–30 sec from the start of the movie sequence it dropped below 0.7 (Fig. 2G). The degrees of co-localization from low to high (as can be seen from the movies and Fig. 2) in lamellipodia were: Myosin1E.paxillin<Myosin1E.β3-integrin<Myosin 1E.CARMIL<Myosin 1E.FHOD. It was possible that Myosin 1E transported CARMIL on actin filaments polymerized from FHOD1 clusters at early adhesion sites and that supported further adhesion formation. CARMIL's interacting partner was Arp2/3 (Jung et al., 2001). We found that Arp3 co-localized with these actin rich transient spots (supplementary material Fig. S5A,B) and may have been involved in polymerization of actin from these spots. Since Myosin1E was concentrated rapidly at the sites of actin filament assembly, then it may have been actively transported to those sites on the actin filaments that assembled there.

### Myosin 1E aggregates move actively in lamellipodia but not Myosin 1G

**Fig. 3.**
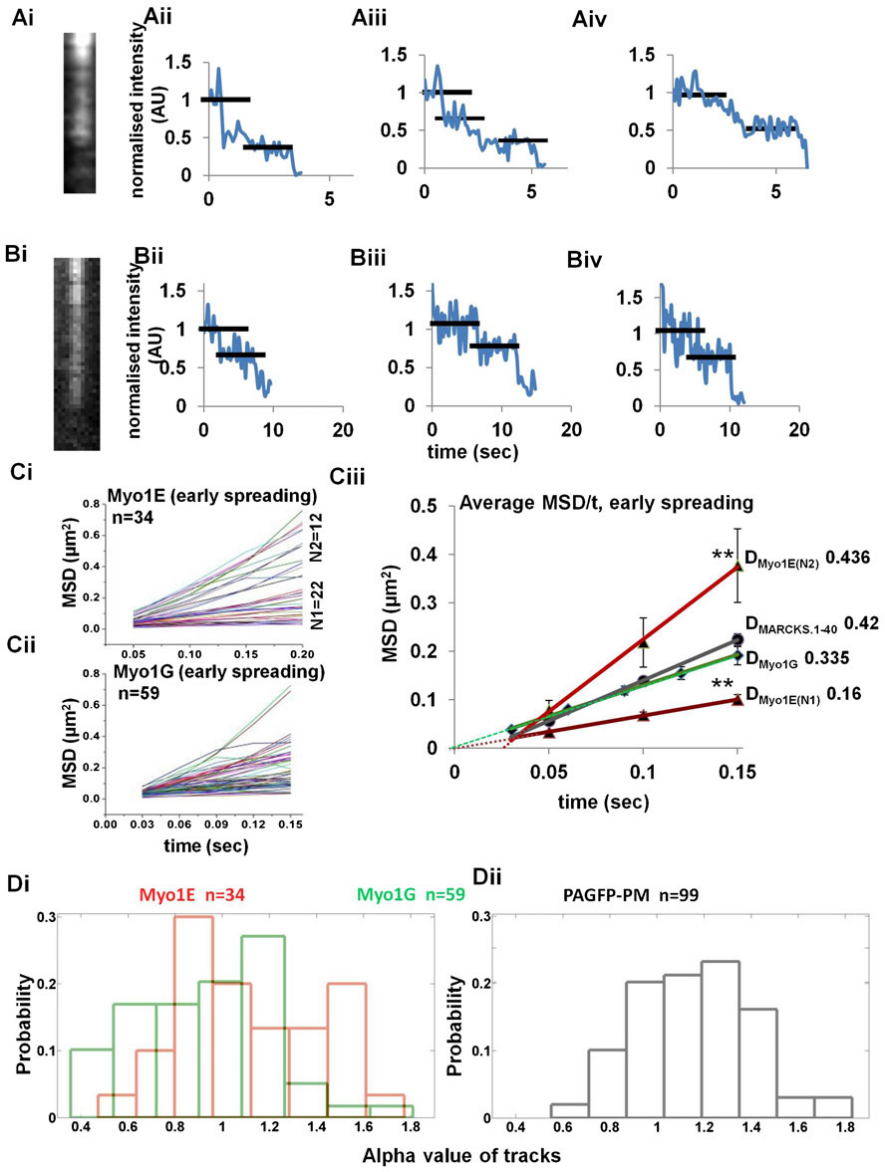
Single particle analysis of Myosin 1E and Myosin 1G oligomers. (A) Bleaching of mApple-Myosin 1E (Ai–Aiv)/EGFP-Myosin1G (Bi–Biv) particles observed in TIRF (formaldehyde–glutaraldehyde fixed sample). Examples shown have two or three bleaching steps (black bars, intensity were normalized to the original observations). (Ci) Summary of all MSD/time plots of Myosin 1E tracks in lamellipodial regions as selected in supplementary material Movie 7. Myosin 1E has two distinct group of tracks, N1 = 22, N2 = 12, total 34 tracks screened. (Cii) Summary of all MSD/time plot of Myosin 1G tracks in lamellipodial regions as selected in supplementary material Movie 8, total 59 tracks screened. (Ciii) Comparative average MSD/time plots of Myosin 1E, Myosin 1G and membrane diffusion control PAGFP-PM. Two groups of Myosin 1E tracks have significantly different average MSD values (*P* = 3.68E−09, at 0.15 sec **). (D) Distribution of alpha-values (as calculated in Materials and Methods by first four time points in logMSD/logtime) of Myosin 1E (red, Di) and Myosin 1G (green, Di) tracks during early spreading. (Dii) PAGFP-PM value distribution is shown as membrane diffusion control.

To determine if Myosin 1E was actively transported in the lamellipodia, we tracked the path of movement of the Myosin 1E molecules. Because Myosin 1s have a low duty ratio and are loosely bound to actin, they are unsuitable for processive movement (De La Cruz and Ostap, 2004; McConnell and Tyska, 2010; Pollard and Ostap, 1996). Based on the, prior studies of long-tailed Myosin 1A/1B from *Acanthamoeba*, it has been suggested that aggregates need to be formed for actin-based motility (Pollard and Ostap, 1996). From plots of the step-bleaching of puncta of Myosin 1E and 1G fluorescence with time (Fig. 3Ai,Bi), it was evident that the puncta contained multiple Myosin 1E and 1G molecules (Fig. 3Aii–iv,Bii–iv) (Pollard and Ostap, 1996). The maximum number of steps observed was 3 and many examples of 2 were seen. When we analyzed the movements of the puncta (supplementary material Movies 7, 8) using a mean-squared displacement versus time plot, we found that the around 1/3 of the Myosin 1E tracks (N2 group) deviated positively from linearity indicating that a fraction of Myosin 1E was actively moving (Fig. 3Ci,iii) and there was another population (N1) that had linear MSD vs time plots and those were considered diffusive. Deviation from positivity of N2 group was marked by the fact that the linear fit of average data-points taken at time t≠0 sec, did not pass through origin (light red dotted line, Fig. 3Ciii) In contrast, Myosin 1G puncta movements generated linear MSD vs time plots that showed no deviation in the majority of tracks (Fig. 3Cii,iii). Both Myosin 1E (N1 group) and Myosin 1G diffusive tracks linear fits, taken identically as before, pass through the origin (dark red and green dotted lines respectively, Fig. 3Ciii). Using the equation MSD = 4Dt+(vt)^2^ (Schmidt et al., 1993) for the Myosin 1E N2 group and plotting various average MSD/t values, given rise to quadratic *y* = 5.14471*x*^2^+1.74578*x*−0.00758071. From here, we calculate that the velocity of active movement of the Myosin 1E puncta was ∼2.26 µm/s and diffusion coefficient D = 0.436 µm^2^/s, ignoring the constant (−0.00758071). Subdiffusive N1 group had D = 0.16 µm^2^/s (Based on fitting equation *y* = 0.666126*x*+0.0004). The Myosin 1G particles had average diffusion coefficient of 0.34 µm^2^/s that was close to the average diffusion coefficient of PAGFP-PM protein (Fig. 1B) fluorescent puncta (0.42 µm^2^/s). Active movement of Myosin 1E was further supported comparing the distribution of alpha values of these tracks with Myosin 1G and PAGFP-PM (using equation logMSD = log4D+αlogt, we plotted logMSD/logt for each individual track. From the slope of each of the lines, we calculated alpha values). We found that Myosin 1G alpha value (Fig. 3Di) distribution had a single peak similar to freely diffusing PAGFP-PM (Fig. 3Dii) (Arbuzova et al., 1998), whereas Myosin 1E alpha values had two peaks, one in subdiffusive range (<1) and one higher than that of Myosin 1G and PAGFP-PM, indicating active motion (Fig. 3Di). Thus, although both Myosin 1E and 1G formed aggregates of 3 or more molecules, only Myosin 1E moved actively in the TIRF field of active lamellipodia. A limitation of above assay is the time duration of observable Myo1E and 1G particles are <0.7 sec on TIRF field (data not shown). Therefore, even with 20–30 fps capture rate, dataset for M.S.D. is of small size. To avoid any artifact due to small dataset, a comparative analysis of Myo1E and 1G is made. Rare unusually long tracks were avoided as those are artifacts.

### Myosin 1E requires multiple domains to localize at the tips of lamellipodia

**Fig. 4.**
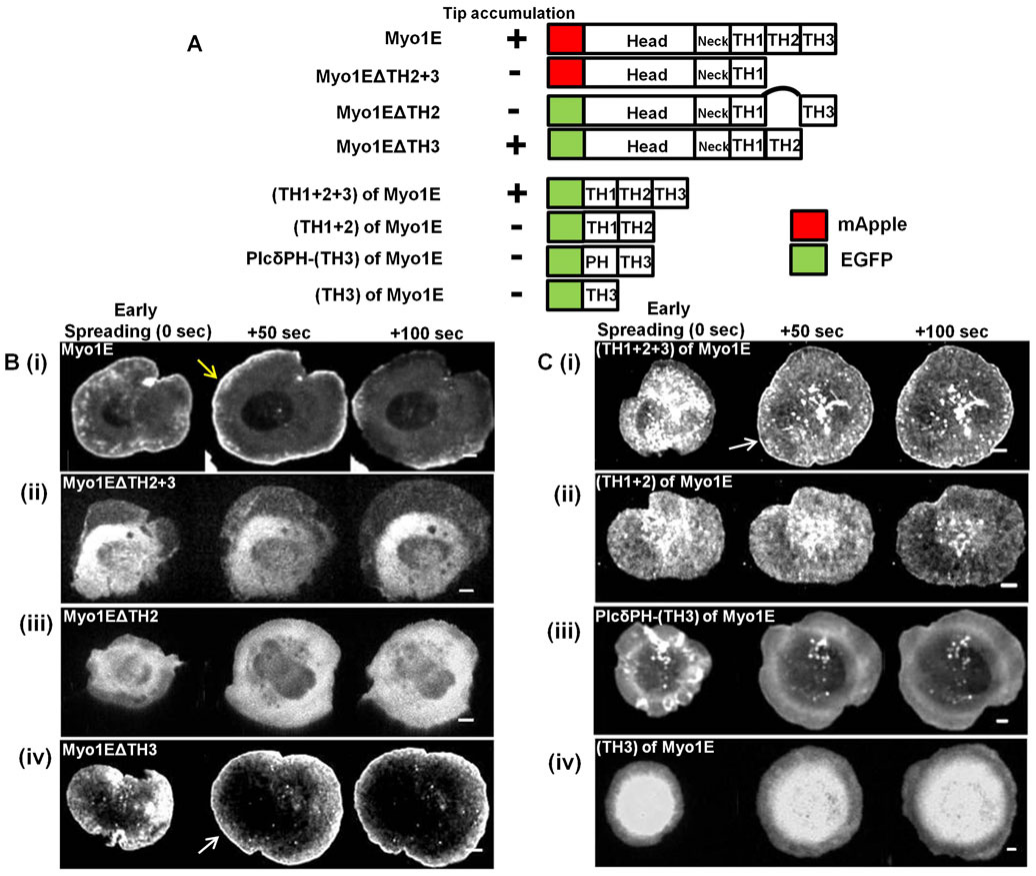
Requirement of multiple domains of Myosin 1E in lamellipodial tip accumulation. (A) Domain map of various myosin 1E deletion (C-terminal and N-terminal) mutants and summary of their lamellipodia tip accumulation during cell spreading process. (B) Edge accumulation of C-terminal deletions. (Bi) Myosin 1E (full length) at the tip of spreading lamellipodia (yellow arrow). No such accumulation in (Bii) Myo1EΔTH2+3 and (Biii) Myo1EΔTH2. Such accumulation was present in (Biv) Myo1EΔTH3 (white arrow). (C) Edge accumulation of N-terminal deletions. (Ci) (TH1+2+3) at the tip of spreading lamellipodia (white arrow). No such accumulation in (Cii) (TH1+2), (Ciii) PlcδPH-TH3 and (Civ) TH3. Bar 5 µm.

To determine which domains of Myosin 1E were involved in lamellipodial tip localization, we designed series of Myosin 1E constructs with both C- and N-terminal deletion mutants (Fig. 4A). We compared tip localization of full-length Myosin 1E (Fig. 4Bi blue arrow) with localizations of each of the deletion mutants in early spreading (0 sec), at the end of fast spreading (50 sec) and in late spreading (100 sec) (Fig. 4B,C). Only Myosin 1EΔTH2+3 (Fig. 4Bii) and Myosin 1EΔTH2 (Fig. 4Biii) failed to localize at the lamellipodia tip. On the other hand, Myosin 1EΔTH3 (Fig. 4A) localized at lamellipodial tip (Fig. 4Biv) as did the tail portion of the molecule TH1+TH2+TH3 (Fig. 4Ci). Myosin 1EΔTH3 and TH1+TH2+TH3 were also found at actin rich early adhesion spots behind the tip of spreading lamellipodia (supplementary material Fig. S6A,B). Because we observed that Myosin 1E formed multimeric complexes, endogenous Myosin 1E SH3 could complex with proline rich TH2 domain of all the constructs that moved to the edge. However, TH2 domain by itself, along with membrane targeting TH1 (TH1+TH2, Fig. 4A), failed to localize at the tip of lamellipodia (Fig. 4Cii). Since TH1+2+3 localized to lamellipodial tip and TH1+2 did not, we investigated role of TH3 domain in tip accumulation. Membrane anchored TH3 (PLCδPH-TH3) or free TH3 domain did not localize to the lamellipodial tip (Fig. 4Ciii,iv). Based upon these findings, we suggest that the oligomerization, of Myosin 1E deletion constructs could enable the aggregates to move to the edge and to bind to F-actin there.

### Expression of Myosin 1EΔTH3 causes lamellipodia to fluctuate in size and orientation

**Fig. 5.**
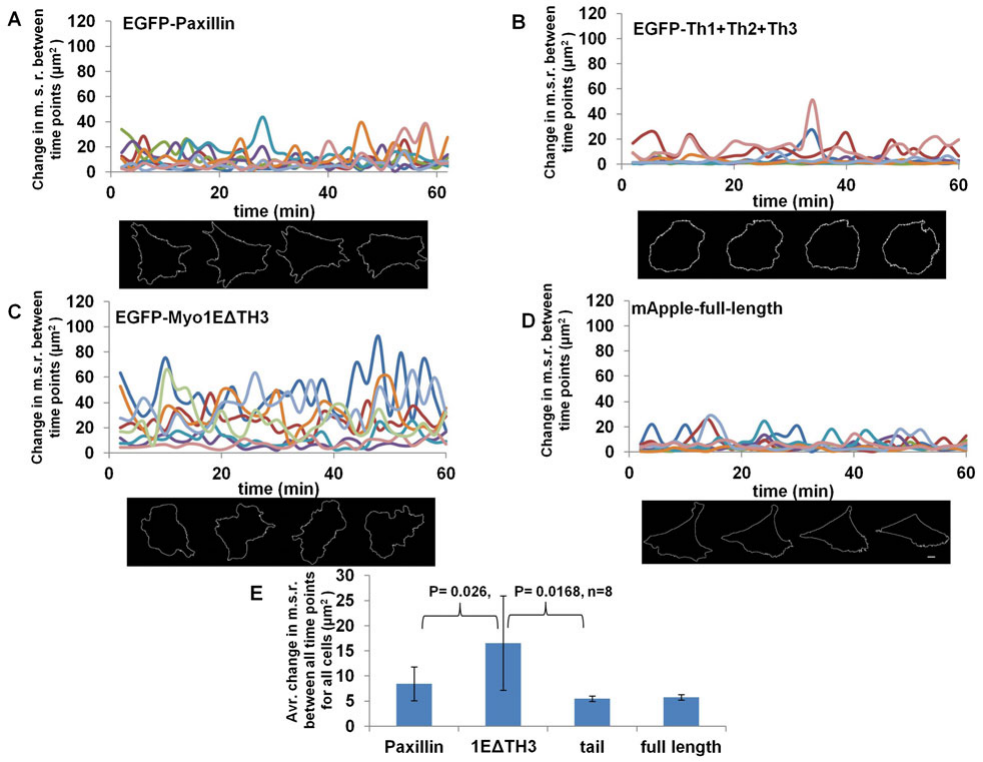
Overexpression of Myosin 1E deletion mutants Myo1EΔTH3 and TH1+TH2+TH3 has opposite effect on lamellipodia dynamics. Tracks of Mean-square radius (M.S.R.) fluctuations between timepoints and one representative cell each for expression of (A) EGFP-Paxillin, (B) EGFP-(TH1+2+3) of Myosin 1E, (C) EGFP-Myosin 1EΔTH3 and (D) mApple-Myosin 1E full length. (E) Average change in M.S.R. per frame (2 sec) of all four constructs (*n* = 8, each). Fluctuation of Myosin 1EΔTH3 is significantly higher from control (*P* = 0.26) and (TH1+2+3) (*P* = 0.0168), were as fluctuation of (TH1+2+3) is lower than control (*P* = 0.046) (supplementary material Movies 9–12). Bar 10 µm.

To relate Myosin 1E localization with function, we expressed TH1+2+3 and Myo1EΔTH3 in cells. Since 1E deletions accumulated at the tip but were potentially not functional, they may have caused changes in the cell/lamellipodia spread-area. We transfected Paxillin-GFP, TH1+2+3 of Myosin 1E, Myosin 1EΔTH3 and full length Myosin 1E, in cells and followed spreading on Fibronectin-coated glass (20× magnification, supplementary material Movies 9–12; Fig. 5). Moderate expression of GFP-Paxillin does not affect lamellipodia (Laukaitis et al., 2001). Paxillin transfected cells polarized after fast spreading and showed very little fluctuation in size thereafter (supplementary material Movie 9). TH1+2+3 transfected cells also behaved like Paxillin cells, but polarization was affected in some cases and cells constricted (supplementary material Movie 10). Myosin 1EΔTH3 transfected cells formed lamellipodia, which constantly fluctuated in length and orientation (supplementary material Movie 11). Fluctuations were not observed in full-length Myosin 1E expressing cells (supplementary material Movie 12), which behaved like control cells. To quantify the fluctuations, time series of mean square fluctuations of the cell radius (M.S.R. fluctuations) [<(δr)2> = Σ(δri)2/N] were computed over all angles from the centroid position, using a custom LabVIEW program (Gupta et al., 2012). Myosin 1EΔTH3, TH1+2+3, full-length Myosin 1E and EGFP-lifeact transfected cells were monitored under identical incubation conditions. At the end of the fast spreading phase (approx. 30 min after spreading), M.S.R. fluctuation data was collected for cells, starting at similar time points. When fluctuations in radius were compared between EGFP-paxillin (Fig. 5A), EGFP-TH1+2+3 (Fig. 5B), EGFP-Myosin 1EΔTH3 (Fig. 5C) and mApple-Myosin 1E (Fig. 5D), we found that Myosin 1EΔTH3 traces showed average M.S.R. fluctuation/frame (16.54 µm^2^) significantly higher than control cells (8.45 µm^2^, *P* = 0.026, *n* = 8). Whereas expression of TH1+2+3 transfected cells lowered the M.S.R. fluctuation from control cells (5.49 µm^2^, *P* = 0.043, *n* = 8, Fig. 5D), which could be explained as an effect on adhesion formation. Expression of full-length Myosin 1E (5.73 µm^2^ Fig. 5D), also showed a reduced M.S.R. fluctuation close to TH1+2+3, but cells showed less constriction at longer times (data not shown). Therefore, the results indicated that there were almost antagonistic roles of the two Myosin 1E deletions in lamellipodia dynamics.

**Fig. 6.**
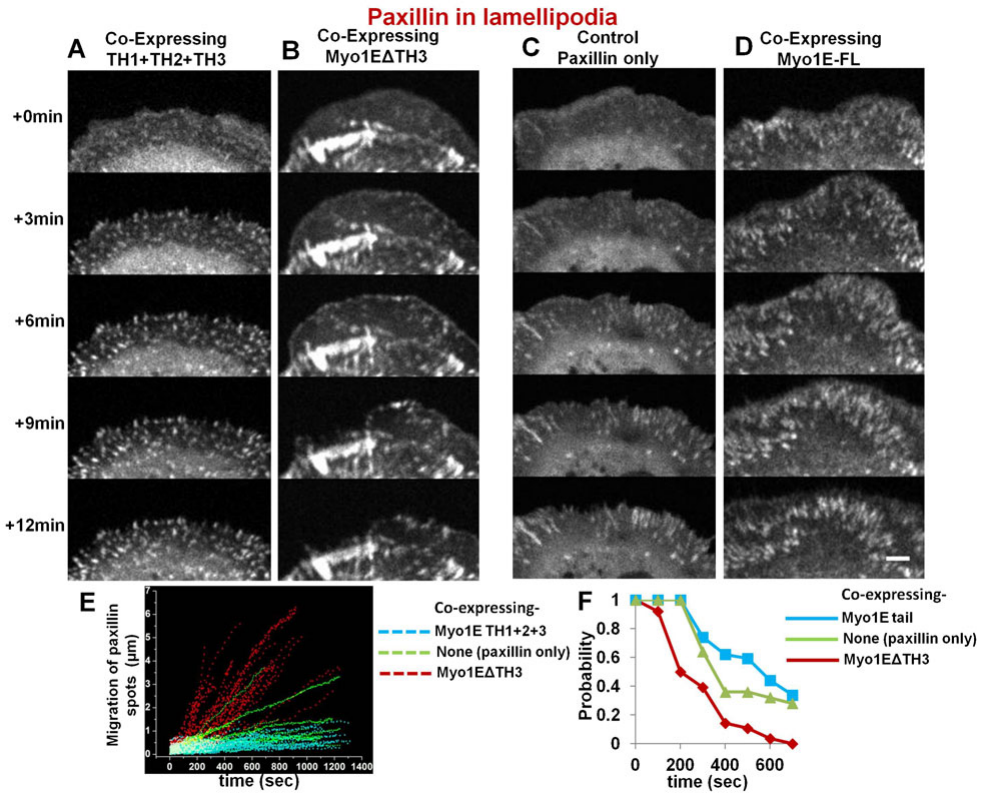
Adhesion formation by paxillin fluorescence: between cells co-expressing full or deletion mutants of Myosin 1E and control cell expressing paxillin alone, showing dominant negative effects of deletion mutants. Dot-like structures of expressed paxillin in lamellipodia of cells co-expressing (A) (Th1+2+3) or (B) Myo1EΔSH3. In (B) newly formed adhesions showed instability in lamellipodia (supplementary material Movie 13). Elongated paxillin structures in active lamellipodia denoting stable control cells expressing paxillin only (C) or co expressing full length Myosin 1E (D). Bar 5 µm. (E) Movement of nascent adhesions in dynamic lamellipodia, as observed by RFP-paxillin spots; observed in cells co-expressing Myo1EΔSH3/(Th1+2+3) and control cells expressing paxillin alone. (F) Probability of finding a paxillin spot within 1 µm radius from where it first appeared in dynamic lamellipodia with time. These nascent adhesions (paxillin-spots) were compared from three cells each (per construct) co-expressing Myo1EΔSH3, (TH1+2+3) or control cells expressing paxillin alone.

To determine if Myosin 1E localization might some way be related to cell adhesion formation, we followed paxillin staining in adhesions of cells expressing Myosin 1E constructs (Fig. 6). We found that in cells expressing Myosin 1E tail or Myosin 1E-ΔSH3 (Fig. 6A,B), the paxillin adhesions were shorter than in control cells expressing paxillin alone (Fig. 6C) or with full-length Myosin 1E (Fig. 6D). We followed adhesions in cells co-expressing paxillin and Myosin 1EΔTH3 or (TH1+TH3+TH3) and compared with control cells expressing paxillin alone. We measured how much movement occurred after adhesion formation (Fig. 6E) and what was the probability they stabilized into a 1 µm radius region around their point of origin (Fig. 6F). In cells expressing Myosin 1EΔTH3, which caused greater fluctuations in cell radius, paxillin adhesions failed to mature and moved dramatically in the dynamic lamellipodia, (Fig. 6E,F; supplementary material Movie 13). The shorter paxillin staining adhesions in those lamellipodia never matured and the lamellipodia retracted. In cells co-expressing (TH1+TH3+TH3), we saw gradual appearance of stable adhesions, similar to cells transfected with paxillin alone (Fig. 6E,F; supplementary material Movie 13). However, adhesions were shorter and more circular. Thus, expression of Myosin 1E domains appeared to affect focal adhesion formation by preventing Myosin 1E interaction with various proteins as proposed above (Fig. 2).

### Myosin 1E knockdown destabilized matrix–lamellipodia interaction

**Fig. 7.**
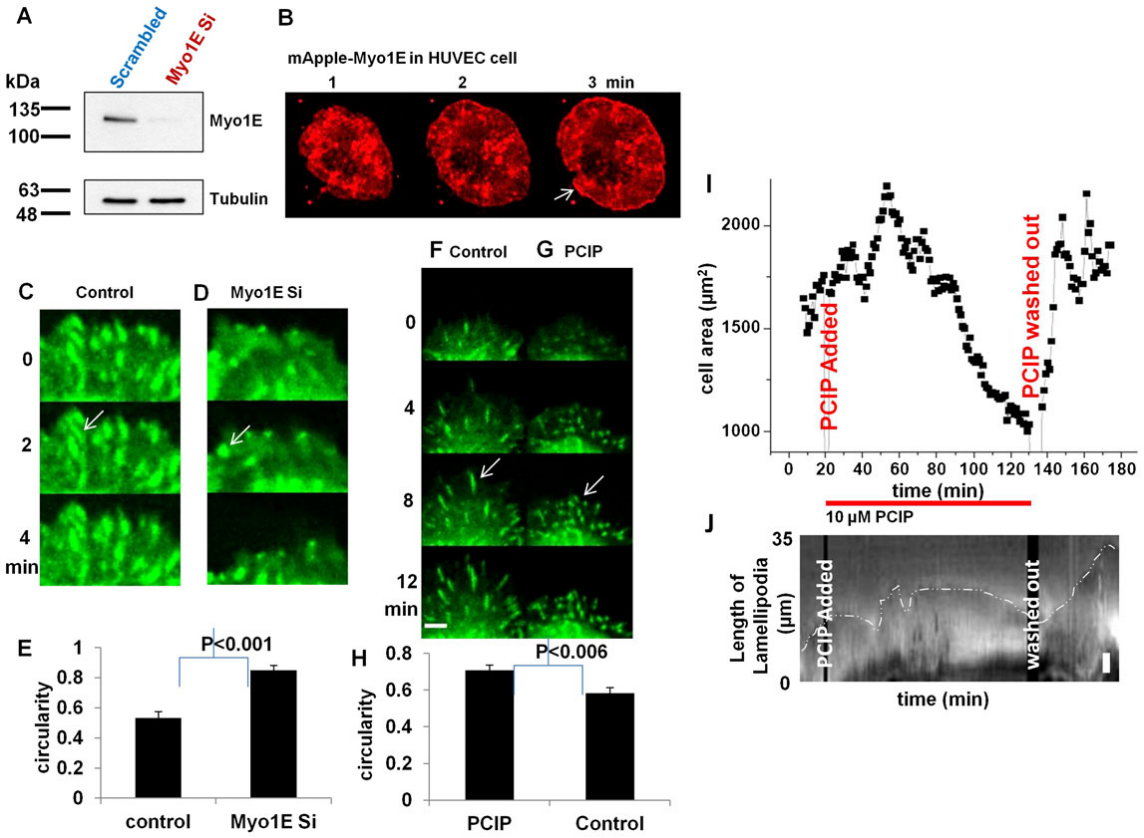
Myosin 1E knockdown has destabilizing effect on cell–matrix adhesions. Similar distablization observed in Myosin 1 inhibitor treated cell. (A) HUVEC cells expressing Myosin 1E, showing edge accumulation (arrow). (B) Knockdown of Myosin 1E in HUVEC cells, (C) Control and (D) Myosin 1E siRNA transfected HUVEC cells (after two consecutive transfections) show difference in circularity of paxillin staining at adhesions (white arrows, supplementary material Movie 14). (E) Myosin 1E siRNA transfected cells have significantly higher circularity than control cells. (F) Control and (G) PCIP treated REF 52-paxillin-GFP cells show difference in circularity of paxillin staining at adhesions (white arrows, supplementary material Movie 15). (H) PCIP treated cells have significantly higher circularity than control cells. (I) Cell area constriction of actively spreading RPTP cells upon addition of PCIP (supplementary material Movie 16). Washout led to re-spreading to same cell area. (J) Kymographic representation of constriction of lamellipodia the cell shown in (I) upon addition of PCIP. Washout led to re-spreading of lamellipodia. Bar 10 µm in A, 5 µm in J, 2 µm in the rest.

To determine whether Myosin 1E depletion affected adhesion formation, we depleted Myosin 1E in HUVEC cells (Fig. 7A). HUVEC cells were chosen because siRNA against human Myosin 1E knocks down >80% Myosin 1E (Cheng et al., 2012). Also, like RPTP, HUVEC generated large lamellipodia when spread on glass and Myosin 1E accumulated at the tips (Fig. 7B white arrow) (Kopp et al., 2010). When Myosin 1E levels were reduced >80%, three days after first siRNA transfection (Fig. 7A), we found no difference in overall or total movement in next 24 hrs of cells transfected with control or Myosin 1E siRNA (data not shown). By expressing GFP-paxillin in Myosin 1E knockdown cells, we followed focal adhesions during spreading and migration. GFP-paxillin in mature focal adhesions was distributed in rod-like structures (Turner et al., 1990; Zaidel-Bar et al., 2003) and those structures were found in two rounds of control siRNA transfected HUVEC cells (Fig. 7C; supplementary material Movie 14). In Myosin 1E knockdown cells, although no visible difference was observed in single round of Myosin 1E siRNA transfection, a second transfection by Myosin 1E siRNA lead to GFP-paxillin concentration in nearly circular bead like structures that were quite dynamic (Fig. 7D; supplementary material Movie 14). There was a significant difference in circularity of the paxillin distributions (across three cells each, Fig. 7E). The lamellipodia of knockdown cells retracted, while those of controls were stable (Fig. 7C,D; supplementary material Movie 14). A similar but more profound effect on adhesion was found after treating the stable paxillin-GFP transfected REF52 cells with global Myosin 1 inhibitor Pentachloropseudilin (PCIP, specific to all isoforms of Myosin 1 only) (Fig. 7F–J; supplementary material Movies 15, 16) (Chinthalapudi et al., 2011). Both control and drug treated cells initially spread-out in a similar way and adhesions were observed in both by paxillin staining (Fig. 7F–G; Movie 15). As time progressed, adhesions of PCIP treated cells remained circular whereas in control cells they elongated (Fig. 7F–G; supplementary material Movie 15). There was a significant difference in circularity in paxillin staining between drug treated and untreated cells, in parallel with knockdown cells (Fig. 7H). The lamellipodia of PCIP treated cells also retracted in similar ways to knockdown cells (Fig. 7F,G; supplementary material Movie 15). The drug treatment was reversible. While addition of the drug constricted the lamellipodia of RPTP cells and reduced cell area, washout restored normal surface area and lamellipodia spreading (Fig. 7I,J; supplementary material Movie 16). Although, a single transfection of Myosin 1E siRNA lowered the protein level by 80%, a second transfection of siRNA was required for an effect on adhesions, which indicated that very low levels of Myosin 1E were needed to see an effect (Fig. 7A). Therefore, global absence in Myosin1 function, might have led to the stronger phenotype such as in PCIP treated cases (Fig. 7F–J). Further, other complementing Myo1 isotypes were also inhibited (Fig. 7F–J).

## Discussion

Based upon these findings there appears to be a major role for Myosin 1E in cell migration and early adhesion formation. Specifically, we find that Myosin 1E accumulates at the tips and at actin-rich sites near early adhesions in actively protruding lamellipodia unlike the short-tailed Myosin 1s (1G, 1B and 1C). Myosin 1E appears to move actively and could transport CARMIL and Arp2/3 complex to sites of actin barbed-end polymerization at the edges of lamellipodia and at very early adhesions with clusters of FHOD1/β3-integrin because a fraction of Myosin 1E actively moves on actin in the TIRF field. Based upon the expression of deletion mutants, accumulation of Myosin 1E at lamellipodial edges requires the membrane binding, TH1 domain with either the head and TH3 domains or the TH2 and TH3 domains. Thus, Myosin 1EΔTH3 and the full tail (TH1+TH2+TH3) constructs localize with Myosin 1E but overexpression of these proteins alters cell function in different ways. Cells expressing Myosin 1EΔTH3 have large lamellipodia that fluctuate rapidly in size and direction. In contrast, cells expressing the full tail have reduced lamellipodial fluctuations. Cells depleted of Myosin 1E still spread lamellipodia rapidly but adhere poorly as evidenced by rapid retraction of lamellipodia and poorly formed dynamic adhesions. A similar phenotype is observed after addition of the global Myosin 1 inhibitor PCIP. Because Myosin 1E could transport CARMIL to FHOD1/β3-integrin sites as well as active leading edges, we suggest that it plays a major role in the transport of components involved in the maturation of adhesions.

Previous studies of Myosin 1E have defined the binding sites for cargo such as, CARMIL and have characterized the motility and membrane binding domains (Feeser et al., 2010; Jung et al., 2001; Krendel et al., 2007; El Mezgueldi et al., 2002). Our finding of multimeric complexes of motile Myosin 1E fits with earlier observations of the aggregation of long tailed Myosin 1E homologs in multimeric complexes (Pollard and Ostap, 1996). The velocity of movement calculated from tracking the actively moving particles was significantly greater than the velocities reported *in vitro* (∼2.26 µm/s *in vivo* vs 0.05 µm/s *in vitro* for rat Myosin 1, (Williams and Coluccio, 1994) and the velocity of actin polymerization from the leading edge (0.1 µm/s from (Giannone et al., 2004). The *in vivo* difference could be due to aggregation and cargo binding that activates Myosin movement (Stöffler and Bähler, 1998). Since Myosin 1E moves toward the barbed end of the actin filaments that would be directed toward active lamellipodial edges and concentrations of the formin, FHOD1, it is logical that Myosin 1E would localize more closely to leading edges and early adhesions (FHOD1 with β3-integrin clusters, (Kiosses et al., 2001)). Similarly, there is a concentration of Myosin 1E at sites of clathrin-dependent endocytosis (Cheng et al., 2012) but these early adhesion structures are not linked to clathrin dependent endocytosis, as they turn-over before clathrin accumulation. Thus, we suggest that Myosin 1E plays an active role in early spreading and motility processes that involve rapid concentration of components at sites of actin polymerization.

The most difficult results to explain relate to the behavior of the mutant forms of Myosin 1E upon overexpression. Localization of the mutant forms can be explained as a result of the oligomerization of proline rich TH2 domain constructs with endogenous Myosin 1E and the loss of oligomerization in the absence of TH2 (Soldati and Kistler, 2004; Stöffler and Bähler, 1998). Oligomerization is a key step for movement and potentially for the binding of cargo. Thus, hetero-oligomers of endogenous 1E with the Myosin 1EΔTH3 should have different properties from those with the full tail (TH1, TH2 and TH3). In the case of Myosin 1EΔTH3 overexpression, there could logically be a deficiency in one of the cargo components that is involved in stabilization of the adhesions and that would result in rapid retractions of the edge, similar to the behavior of Myosin 1E depleted cells. Greater fluctuations with 1EΔTH3 overexpression could result from the motor activity that could support new extensions (unlike the case with 1E depletion) that again would not form stable adhesions. They cannot be due to inhibition of Myosin II driven contraction, since Myosin 1 heads do not bind to tropomyosin decorated actin, where Myosin II binds (Tang and Ostap, 2001). However, the decrease in overall motility with full tail expression could be the result of a decreased activation of Myosin II as well as the decreased motility of the Myosin 1E hetero-oligomers. Actin polymerization at the edge could be compromised by decreased transport and a relative increase in the cargo domain. There are other potential roles for Myosin 1E that could further explain these findings but they are still consistent with a general model that Myosin 1E is transporting important components to the sites of actin polymerization that are involved in stimulating actin polymerization and/or adhesion formation at integrin sites.

In the absence of Myosin 1E, mice are viable as are their fibroblasts; however, the animals have severe loss of podocytes in their kidneys with concomitant loss of kidney function. Since there is another long Myosin isoform, 1F, it may be upregulated to improve cell function. This might be the reason behind weaker phenotype of Myosin 1E knockdown compared to overexpression phenotype of deletion mutants or global Myosin 1 inhibition by PCIP. The loss of podocytes is consistent with a compromise in adhesion formation since podocytes are thin actin-based protrusions that must be stabilized on basement membrane sites. Generally, Myosin 1s have the ability to diffuse as monomers and to move as oligomers. The unusual role of Myosin 1E in concentrating to sites of actin polymerization in early spreading cells indicates that it is particularly active in that cell environment. Other Myosin 1s may be upregulated for transport when adhesions are stabilized or under steady state conditions. Upregulation of Myosin 1F or other form of Myosin 1 isotypes in absence of Myosin 1E might lead to survival of knockout mice. This hypothesis is supported by the fact that inhibition of all Myosin 1 isotypes by PCIP had a more profound effect on cell matrix adhesion than Myosin 1E depletion alone. Taken together, we propose that Myosin 1E is involved in actin organization in active lamellipodia and that leads to formation of early adhesions.

## Materials and Methods

### DNA constructs and antibody

The constructs for pEGFPC1-Myosin1G (Olety et al., 2010) was kind gifts of Prof. Martin Bähler. pUHD10-3-Myosin 1E (RAT) was a gift from Prof. Ed Manser. Human EGFP-CARMIL1a was a gift from Prof J. Cooper (Yang et al., 2005). GFP-tagged Myosin 1E tail construct (TH1+TH3+TH3) was previously described (Krendel et al., 2007). PM-GFP (GFP-pcDNA3-LYN with membrane targeting Lyn (MGCIKSKRKD) was generated by Prof. Tobias Meyer and was purchased from ADDgene. Other GFP-tagged Myosin 1E deletion constructs were generated using In-Fusion HD PCR cloning (Clontech). pYFPC1-Myosin1C was a kind gift of from Prof. Michael Czech (Bose et al., 2002). pmApple-Myosin 1E (Taylor et al., 2011) was obtained from the authors via ADDGENE. DNA construct containing Myosin1F (ATCC clone MGC-40199, I.M.A.G. clone id-5213035) and Myosin 1B (ATCC No-10699052, I.M.A.G. clone id-6821232 and ATCC No-10469400, I.M.A.G. clone id-6487332) were purchased from BioRev/BioGen®. For making Myosin 1E deletion, we followed the domain map as previously described (Krendel et al., 2007). All Myosin 1 (full-length or deletion) open reading frames that are mobilized to PAmcherryC1 (Subach et al., 2009) were cloned using circular PCR method. PAGFP-PM is (MARCKS, 1–40 residues that contained membrane insertion palmytoylation sites at residue 3 and 4) (De Paola et al., 2003). Myosin 1E antibody was identically used as described (Skowron et al., 1998).

### Cell cultures and Myosin 1 inhibitor

RPTPα^+/+^ cells (Sap et al., 1990; Su et al., 1999) were cultured in GIBCO DMEM high glucose (11965, includes phenol red), with 10% fetal bovine serum, 100 U/ml penicillin, 100 µg/ml streptomycin and 1 mM sodium pyruvate. For imaging, DMEM high glucose (21041, without phenol red) was used with all above mentioned additives.

HUVEC cells (Yamada et al., 1992) were cultured in fully supplemented EGM media (Invitogen), following media instructions.

Ref52-YFP paxillin cells (Zaidel-Bar et al., 2007) were available as lab stock and treated in the same way as RPTP cells above.

Myosin 1 inhibitor PCIP (Pentachloropseudilin) (Martin et al., 2009; Chinthalapudi et al., 2011) was dissolved in DMSO in a 25 mM stock and added at a final concentration of 10 µm at the beginning or in between timepoints as given in figure captions.

### Transfection of DNA and siRNA

Approximately 0.5×10^6^ RPTP cells were transfected with 5 µg PAmcherry/mApple-Myosin 1 constructs+0.5 µg EGFP-lifeact, using 100 µl electroporation tips by a Neon™ electroporator, following manufacturer's instructions (1700V, 20 ms, 1 pulse). For siRNA transfection to HUVEC cells, 10 µl electroporation tips and 1 µg siRNA (Dharmacon smart pool) was used (1200v, 40 ms, 1 pulse). For GFP-paxillin observation in knockdown cells, a second transfection with 1 µg siRNA+1 µg DNA was done and incubated for additional two days.

### Confocal microscopy

#### Photoactivation

After overnight incubation of transfected cells at 37°C+5% CO_2_, cells were freshly split into a Greiner™ 35 mm glass bottom dish (fibronectin coated) in imaging medium and visualized in a PerkinElmer™ Spinning Disk confocal (Built on Olympus IX inverted microscope, 100× PL FL oil lenses of N.A. 1.4, Hamamatsu C9100-13 512×512 pixel back illuminated EMCCD camera). Imaging media was DMEM(−) phenol red(−) serum, imaging was done in 37°C+5% CO_2_. EGFP-lifeAct fluorescence was used to find transfected cells and see the actin structure. Photo-activation of PAmcherry in region of activation (ROA) and observation of the active molecules were carried out using the UltraVIEWPhotoKinesis™ module, set with the following protocol: Pre-bleach images were captured by 561 nm laser excitation for 2 seconds. Photo-activation was by 405 nm laser using UltraVIEW PK Device as a bleaching device for 0.2 to 1.0 s. Post-activation images were recorded for 5 seconds at the maximum rate. Shutters wereset for maximum speed. The 405 nm activation laser was set at maximum power, 561 laser at 50% of the maximum power.

#### Time course confocal observation

For observing mApple-Myosin 1E and/or EGFP-deletion constructs custom made spinning disk microscope built on Nikon Eclipse Ti was used. Images were captures every 2 sec in a photometrics Cascade II EMCCD camera by 60× oil lance (N.A. 1.4) until required.

### TIRF microscopy

Cells were identically processed as before for microscopy and visualized in an Olympus TIRF microscope (Olympus IX inverted microscope, 100× PL FL TIRF oil lenses (N.A. 1.45), Photometrics Cascade II 1024×1024 EMCC camera). The 561/488 nm (in epi and TIRF) visualization were done using single camera every 1/2 sec. 100 nm red/green beads were used to align multi-channel data.

### Bleaching of EGFP-Myosin1G/mApple-Myosin 1E in TIRF

RPTP cells were transfected with 2 µg EGFP-Myosin1G/2 µg mApple-Myosin 1E DNA by Neon™ electroporator, following manufacturer's instructions. Before observation, cells were fixed in 4% paraformaldehyde+0.1% glutaraldehyde. Sample processing methods were the same as used in the photo-activation experiments. Cells were observed in the Zeiss Elyra microscope under 60–100% of 488 nm laser power in TIRF and with maximum EM gain for 200 ms exposure time (100× TIRF lenses).

### Tracking Myosin 1E/1G clusters in TIRF layer of lamellipodia

For tracking Myosin 1E and Myosin 1G as moving clusters, maximum-power of lasers was applied to make them visible. One general observation was made that most of the Myosin 1E particles stays in about 5 µm from the cell edge during P1 spreading, roughly covering the width of lamellipodia from cell edge. Therefore, given the thickness of lamellipodia (100–200 nm, (Atilgan et al., 2005)) is negligible, we considered 2D motions only. For particle tracking, boxed regions covering lamellipodia (as shown in supplementary material Movies 3–6) are selected and cropped out of the main movie. It was rotated (clockwise in this case) so that lamellipodial edge remains horizontal and parallel to the top edge of the box. Then it was fed into imaris software and origin of reference was chosen at the bottom left corner of the box. To make comparison uniform, similar regions were cropped out for Myosin 1G and PAGFP-PM particle also. Although in these cases, distributions of particles were more uniform in TIRF layer. For particle tracking, connected components were only considered to avoid false tracking. Diameters of these particles were approx. 0.5 µm and movie speed was 20 (Myosin 1E) or 30 (Myosin 1E) fps. Therefore any particles having instantaneous speed >10 µm/s was not considered. Also track-length of >10 timepoints were only considered as smaller tracks would not be useful in MSD calculations. To eliminate frame to frame noise, particles that have total displacement of at least their diameter length were considered. *x*, *y* coordinates of each point in tracks was exported and MSD/time plotted. D of particles was calculated from first three time points using linear fit, http://people.hofstra.edu/Stefan_Waner/RealWorld/newgraph/regressionframes.html. Only the slope of the average linear fits were considered for calculating D and used for close linear fits of Myosin 1G and PAGFP-PM. For nonlinear fits, quadratic equation was used, as described (Schmidt et al., 1993).

To investigate presence of super diffusive nature in Myosin 1G or 1E tracks, the following formula was used.
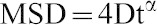

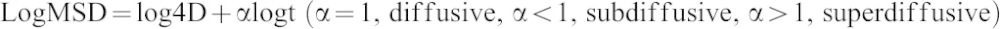
Individual alpha values of each track was calculated from logMSD vs logt plot and plotted as histogram.

### DIC/phase contrast/fluorescence observation of Myosin 1E knockdown and Myosin 1E deletion overexpressing cells

HUVEC cells were resuspended in 1× Ringer medium and spread on 10 µg/µl fibronectin coated glass slides. Observation was on an Olympus Live microscope by 20× PL FL objective for four hours (N.A. 0.45), every 2 min interval.

Spreading of EGFP-Myosin 1E deletion constructs were carried out on on 10 µg/µl fibronectin coated plastic dish in Nikon Biostation IMQ microscope, by 20× air lenses (N.A. 0.5). Transfected cells were spread at identically maintained Biostation IMQ microscope and allowed them to undergo spreading. When the fast spreading was visually over (approx. 30 min after spreading), data were collected for cells, starting at similar time points.

### Image processing

Volocity (Spinning disk confocal) and MetaMorph (Olympus TIRF and Elyra) softwares were used with respective microscopes. Image processing was carried out by ImageJ (NIH, USA), aided by various plugins.

Data analysis was carried out by Microsoft Excel, Origin and in Matlab. For measuring Mean Square cell radius (M.S.R.) fluctuations, cell area measurement was done thresholding the fluorescence of overexpressing constructs. Cell boundary then obtained by “analyze particle” plug-in of ImagJ. Cell boundary information then input into published programs for M.S.R. fluctuations (Bhattacharya et al., 2009; Gupta et al., 2012). Other programs were custom written.

## Supplementary Material

Supplementary Material
