# Supplementary material for: Myosin 1E localizes to actin polymerization sites in lamellipodia, affecting actin dynamics and adhesion formation
Source: Biol Open. 2013 Oct 16;2(12):1288–99. doi: 10.1242/bio.20135827 (PMC3863413; doi:10.1242/bio.20135827)
Supplement: Supplementary Material [file supp_2_12_1288__index.html]

Myosin 1E localizes to actin polymerization sites in lamellipodia, affecting actin dynamics and adhesion formation — Supplementary Material 

# Myosin 1E localizes to actin polymerization sites in lamellipodia, affecting actin dynamics and adhesion formation

## bio.20135827 Supplementary Material

**Files in this Data Supplement:**

- Supplementary Material - Prabuddha Gupta et al. doi: 10.1242/bio.20135827
- Movie 1 - **Movie 1.Gradual accumulation of mApple-Myosin 1E (red) at the tip of lamellipodia of a spreading RPTP fibroblast cell.** Co-expressed PM-GFP is shown in green and cell contour in DIC. 30 frames per minute (fpm) images were collected by 60× lenses of a Yokogawa confocal spinning disk microscope (based on a Nikon Ti system) and played at 20 fps. Bar 5 µm.
- Movie 2 - **Movie 2. Periodic appearance of Myosin 1E (red) at the tip of lamellipodia in TIRF layer.** Myosin 1E is also co-localized with actin rich spots (as seen by green lifeact) in the lamellipodia. Filmed by 100× lenses of an Olympus XI based iLas TIRF microscope at 30 fpm and played at 20 fps. Bar 2 µm.
- Movie 3 - **Movie 3. Localization of mApple-Myosin 1E and EGFP-β3-integrin in the TIRF layer of spreading lamellipodia.** Myosin 1E co-localizes with β3-integrin during cell spreading. Supplementary material Movies 3–6 identically captured and played as supplementary material Movie 2. Bar 5 µm.
- Movie 4 - **Movie 4. Localization of mApple-Myosin 1E and FHOD1 in the TIRF layer of spreading lamellipodia.** Myosin 1E co-localizes with FHOD1 during cell spreading. Supplementary material Movies 3–6 identically captured and played as supplementary material Movie 2. Bar 5 µm.
- Movie 5 - **Movie 5. Localization of mApple-Myosin 1E and CARMIL1 in the TIRF layer of spreading lamellipodia.** Myosin 1E co-localizes with CARMIL 1 during cell spreading. Supplementary material Movies 3–6 identically captured and played as supplementary material Movie 2. Bar 5 µm.
- Movie 6 - **Movie 6. Localization of mApple-Myosin 1E and Paxillin in the TIRF layer of spreading lamellipodia.** Co-localization of Myosin 1E and Paxillin is not visible during cell spreading. Supplementary material Movies 3–6 identically captured and played as supplementary material Movie 2. Bar 5 µm.
- Movie 7 - **Movie 7. Single particle movement of PAmcherry-Myosin 1E captured by an 100× Olympus IX inverted microscope based TIRF imaging system at 20 fps.** Maximum power 561 nm laser and maximum possible gain was used to make the moving particles visible. Movie was played at real time. Bar 5 µm.
- Movie 8 - **Movie 8. Single particle movement of PAmcherry-Myosin 1G captured by an 100× Olympus IX inverted microscope based TIRF imaging system at 30 fps.** Maximum power 561 nm laser and maximum possible gain was used to make the moving particles visible. Movie was played at real time. Bar 5 µm.
- Movie 9 - **Movie 9. Observation of cells for 1 hr in 20× mag, stating from approx. 30 min from attaching to 10 µg/ml fibronectin coated surface.** EGFP-Paxillin transfected cells were imaged every 2 min in a Nikon Biostation IMQ microscope. Movie was played at 20 fps. Bar 10 µm.
- Movie 10 - **Movie 10. Observation of cells for 1 hr in 20× mag, stating from approx. 30 min from attaching to 10 µg/ml fibronectin coated surface.** TH1+2+3 transfected cells were imaged every 2 min in a Nikon Biostation IMQ microscope. Movie was played at 20 fps. Bar 10 µm.
- Movie 11 - **Movie 11. Observation of cells for 1 hr in 20× mag, stating from approx. 30 min from attaching to 10 µg/ml fibronectin coated surface.** Myo1EΔTH3 transfected cells were imaged every 2 min in a Nikon Biostation IMQ microscope. Movie was played at 20 fps. Bar 10 µm.
- Movie 12 - **Movie 12. Observation of cells for 1 hr in 20× mag, stating from approx. 30 min from attaching to 10 µg/ml fibronectin coated surface.** mApple-Myosin 1E transfected cells were imaged every 2 min in a Nikon Biostation IMQ microscope. Movie was played at 20 fps. Bar 10 µm.
- Movie 13 - Movie 13. (A) RFP-Paxillin and co-expressing EGFP-Myosin 1EΔSH3 (inset), (B) RFP-Paxillin and co-expressing EGFP-(TH1+2+3) (inset), observed in Yokogawa confocal spinning disk microscope (based on a Nikon Ti system) every 2 sec by 60× lenses and played at 100 fps. Bar 5 µm.
- Movie 14 - **Movie 14. GFP-Paxillin patterns in contracting lamellipodia (imaged every 3 min, played at 20 fps), in scrambled siRNA transfected cells (left) and Myosin 1E transfected siRNA cells (right).** Myosin 1E depletion made adhesions weak and paxillin staining round shaped. Bar 2 µm.
- Movie 15 - **Movie 15. GFP-Paxillin patterns in contracting lamellipodia (imaged every 4 min, 20 fps), in Myo1 inhibitor PCIP treated cells (right) and untreated cells (left).** Myosin 1E depletion made adhesions weak and paxillin staining round shaped. Bar 2 µm.
- Movie 16 - **Movie 16. Two cells were allowed to spread and PCIP was added at 20 min and washed out at 130 min.** Imaged every minute, played at 20 fps. Bar 5 µm.
