# Supplementary material for: Myosin 1E localizes to actin polymerization sites in lamellipodia, affecting actin dynamics and adhesion formation
Source: Biol Open. 2013 Oct 16;2(12):1288–99. doi: 10.1242/bio.20135827 (PMC3863413; doi:10.1242/bio.20135827)
Supplement: Supplementary Material [file supp_bio.20135827_bio.20135827-s1.pdf]

Supplementary Material

Prabuddha Gupta et al. doi: 10.1242/bio.20135827

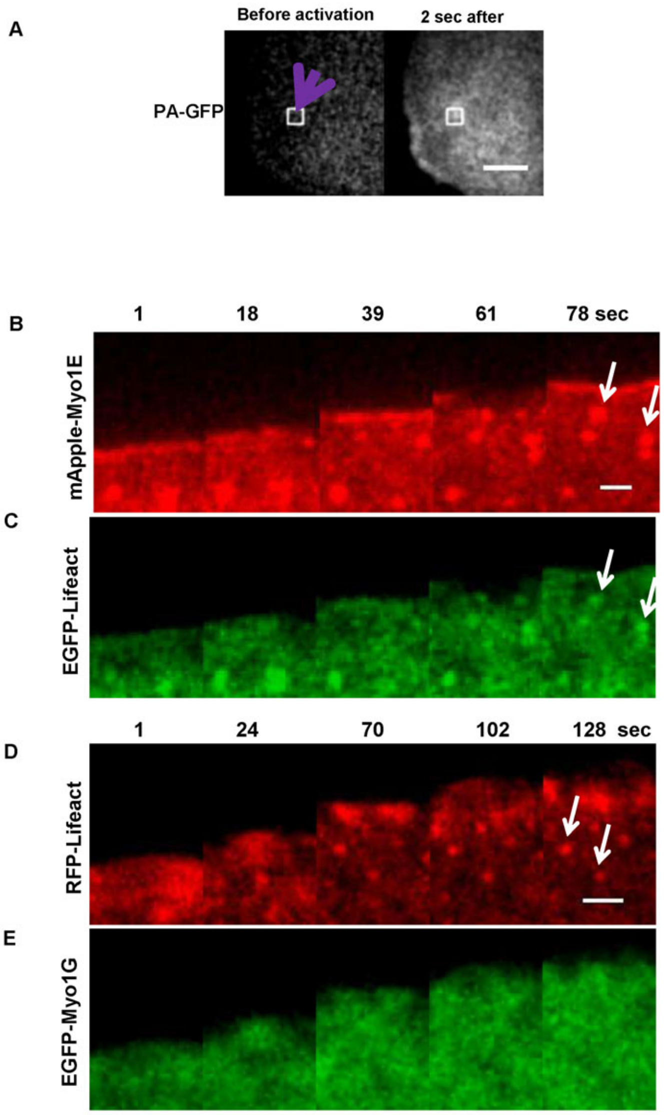

Fig. S1. Myosin 1E but not Myosin 1G co-localize with actin rich spots in the ventral layer of lamellipodia. (A) Photoactivation of a cell transfected with PA-GFP by 405 nm laser (purple arrow, 0–2 sec), observed by 488 nm laser excitation. Bar 5 µm. Stationary dot like structures as it appear in TIRF layer (B) in Myosin 1E channel in spreading cell and presence (C and D) of similar co-localized dots in lifeact-GFP/RFP channel. (E) Myosin 1G shows no such dots that could co-localize with actin spots. Bar 2 µm.

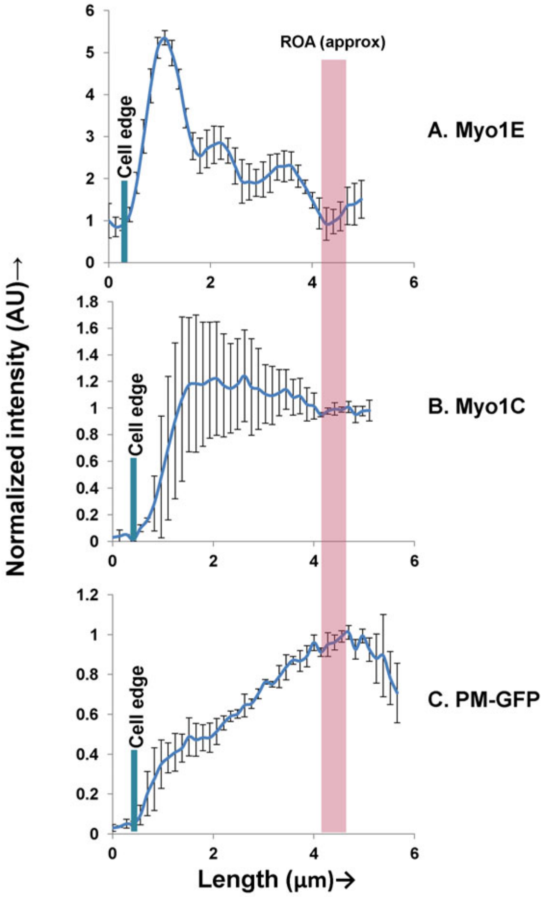

Fig. S2. Myosin 1E uniquely accumulate at the tip of lamellipodia when activated from a ROA approx. 4 µm back from tip of lamellipodia. This figure is a repetition of Fig. 1G to show reproducibility of data, showing average of three cells for each constructs, taken in independent experiments. (A) PAmCherry-Myo1E, (B) PAmcherry-Myo1C and (C) PAGFP-PM intensity profile after two second of photo-activation from the ROA. Location of ROA is shown as violet bar.

Biology Open

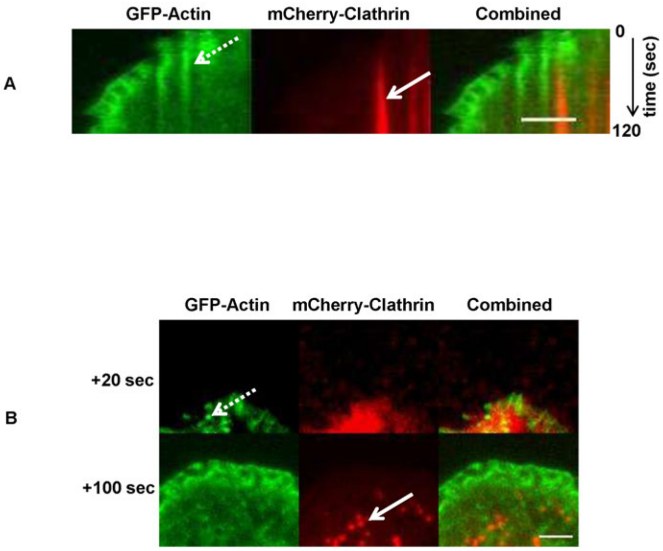

Fig. S3. Actin rich early adhesion spots in lamellipodia is spatially and temporally distinct from clathrin rich punctuate structures. (A) Kymograph from a spreading cell in TIRF microscope showing GFP-Actin rich spot (dotted arrow) is spatially and temporally independent from mCherry-clathrin rich spots (filled arrow) and precedes the later. (B) Still image from the same spreading process indicating actual location of such spots during the time-course. Dotted arrow for actin and filled arrow for clathrin. Bar 5 μm.

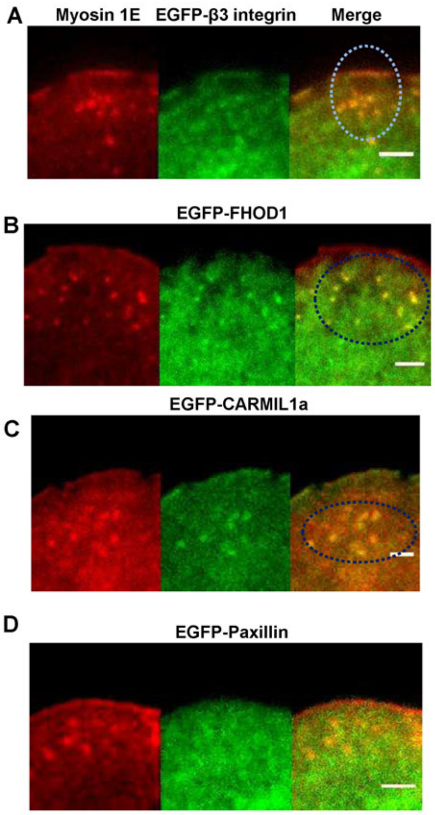

Fig. S4. Stills from supplementary material Movies 3–6 sequences. (A) Myosin 1E and β3-integrin, (B) Myosin 1E and FHOD1, (C) Myosin 1E and CARMIL1a and (D) Myosin 1E and Paxillin. Bar 5 μm.

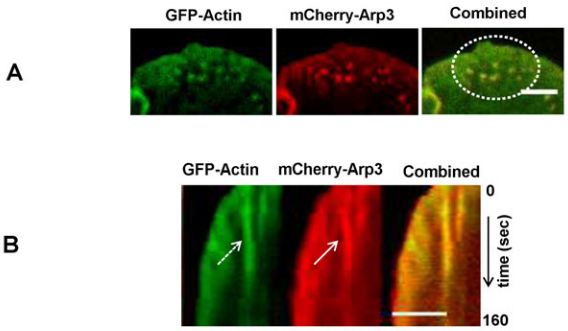

Fig. S5. Actin rich early adhesion spots in lamellipodia co-localize with Arp3. (A) Still image from cell spreading in a TIRF microscope, cell expressing GFP-actin and mCherry-Arp3, showing region of lamellipodia were actin rich spots co-localize with Arp3 spots. (B) Kymograph from the spreading cell, showing Actin rich spot (dotted arrow) co-localize with Arp3 spots (filled arrow). Bar 5 μm.

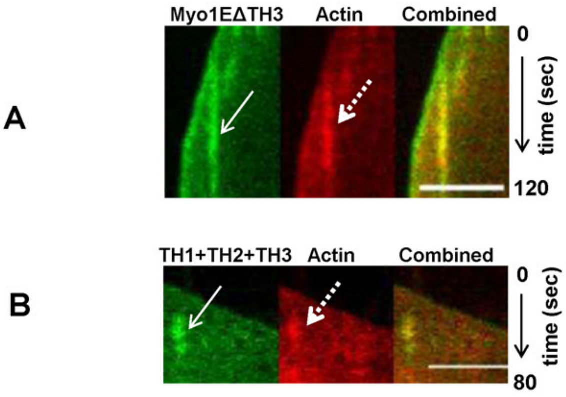

Fig. S6. Actin rich early adhesion spots in lamellipodia co-localize with Myo1EΔTH3 and TH1+TH2+TH3. (A) Kymograph from the spreading cell showing RFP-Actin rich spot (dotted arrow) co-localize with EGFP-Myo1EΔTH3 (filled arrow). (B) Kymograph from the spreading cell showing RFP-Actin rich spot (dotted arrow) co-localize with EGFP-TH1+TH2+TH3 (filled arrow). Bars 5 μm. Both movies were taken by spinning disk microscope.

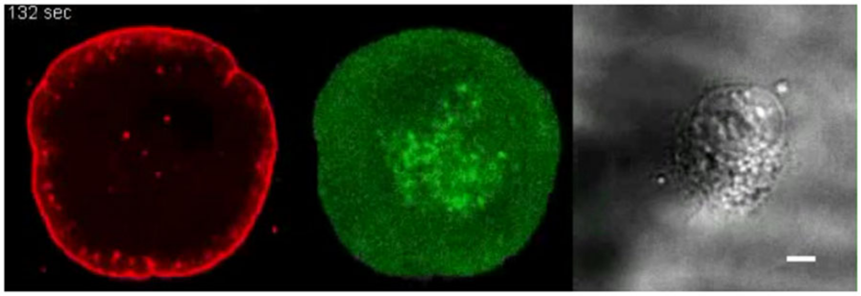

Movie 1. Gradual accumulation of mApple-Myosin 1E (red) at the tip of lamellipodia of a spreading RPTP fibroblast cell. Co-expressed PM-GFP is shown in green and cell contour in DIC. 30 frames per minute (fpm) images were collected by 60× lenses of a Yokogawa confocal spinning disk microscope (based on a Nikon Ti system) and played at 20 fps. Bar 5 μm.

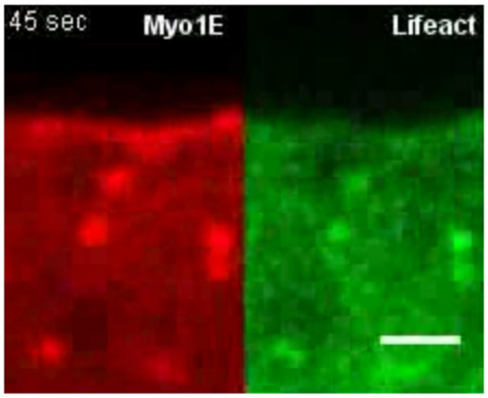

Movie 2. Periodic appearance of Myosin 1E (red) at the tip of lamellipodia in TIRF layer. Myosin 1E is also co-localized with actin rich spots (as seen by green lifeact) in the lamellipodia. Filmed by 100× lenses of an Olympus XI based iLas TIRF microscope at 30 fpm and played at 20 fps. Bar 2 μm.

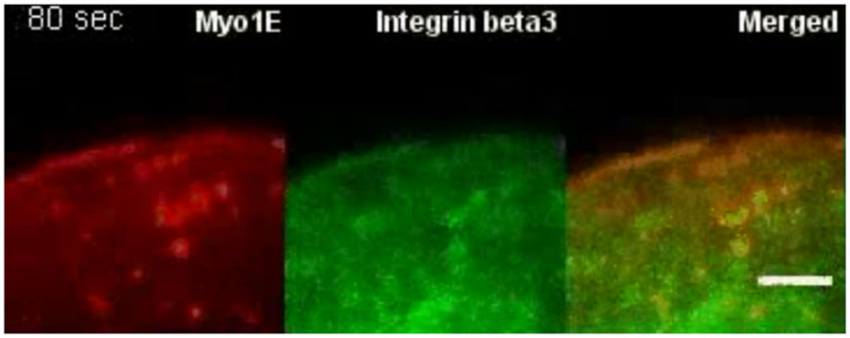

Movie 3. Localization of mApple-Myosin 1E and EGFP-β3-integrin in the TIRF layer of spreading lamellipodia. Myosin 1E co-localizes with β3-integrin during cell spreading. Supplementary material Movies 3–6 identically captured and played as supplementary material Movie 2. Bar 5 μm.

Biology Open

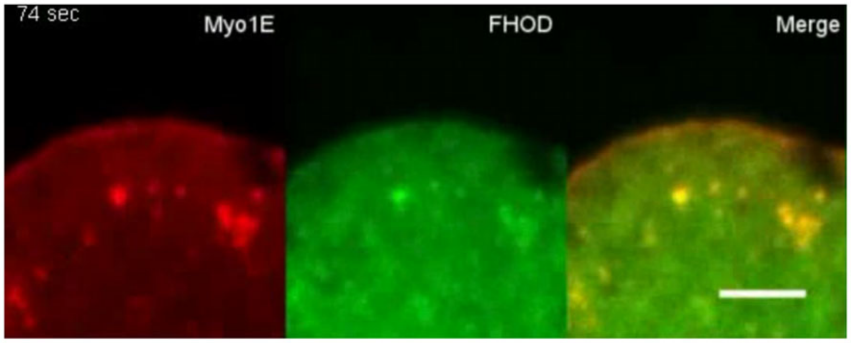

Movie 4. Localization of mApple-Myosin 1E and FHOD1 in the TIRF layer of spreading lamellipodia. Myosin 1E co-localizes with FHOD1 during cell spreading. Supplementary material Movies 3–6 identically captured and played as supplementary material Movie 2. Bar 5 µm.

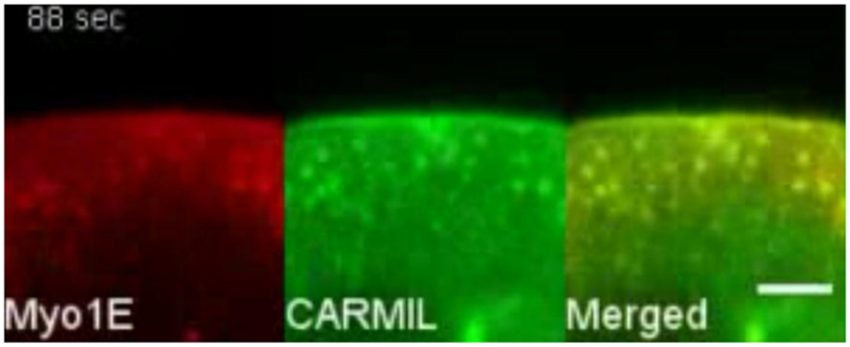

Movie 5. Localization of mApple-Myosin 1E and CARMIL1 in the TIRF layer of spreading lamellipodia. Myosin 1E co-localizes with CARMIL 1 during cell spreading. Supplementary material Movies 3–6 identically captured and played as supplementary material Movie 2. Bar 5 µm.

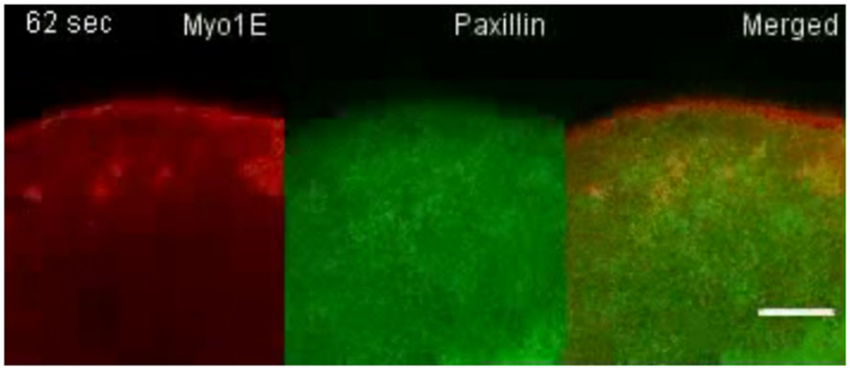

Movie 6. Localization of mApple-Myosin 1E and Paxillin in the TIRF layer of spreading lamellipodia. Co-localization of Myosin 1E and Paxillin is not visible during cell spreading. Supplementary material Movies 3–6 identically captured and played as supplementary material Movie 2. Bar 5 µm.

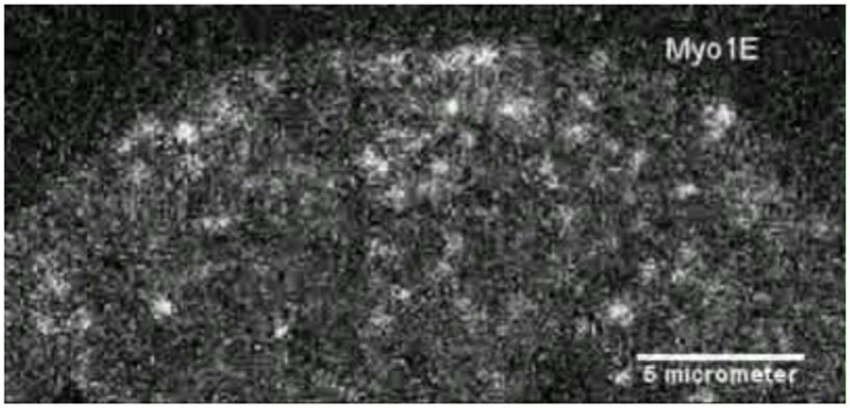

Movie 7. Single particle movement of PAmcherry-Myosin 1E captured by an 100× Olympus IX inverted microscope based TIRF imaging system at 20 fps. Maximum power 561 nm laser and maximum possible gain was used to make the moving particles visible. Movie was played at real time. Bar 5 µm.

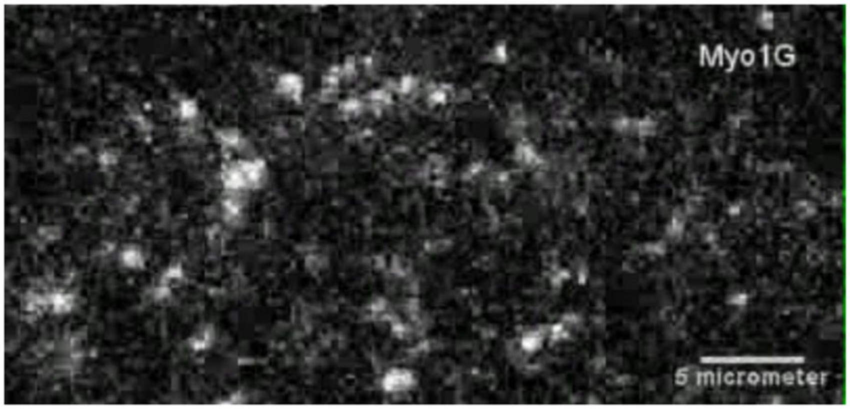

Movie 8. Single particle movement of PAmcherry-Myosin 1G captured by an 100× Olympus IX inverted microscope based TIRF imaging system at 30 fps. Maximum power 561 nm laser and maximum possible gain was used to make the moving particles visible. Movie was played at real time. Bar 5 µm.

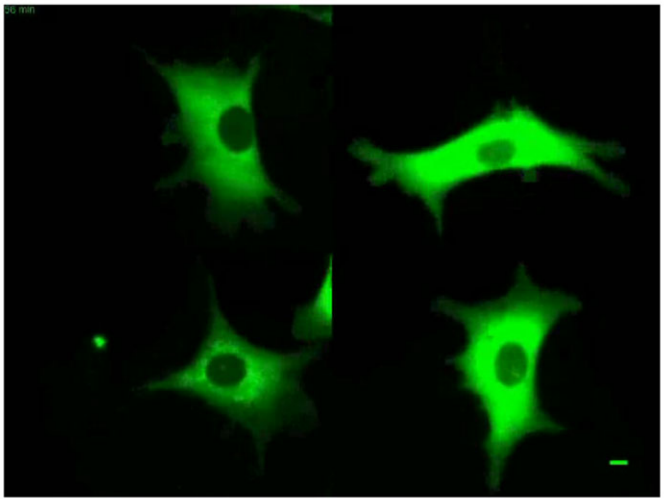

Movie 9. Observation of cells for 1 hr in 20× mag, stating from approx. 30 min from attaching to 10 µg/ml fibronectin coated surface. EGFP-Paxillin transfected cells were imaged every 2 min in a Nikon Biostation IMQ microscope. Movie was played at 20 fps. Bar 10 µm.

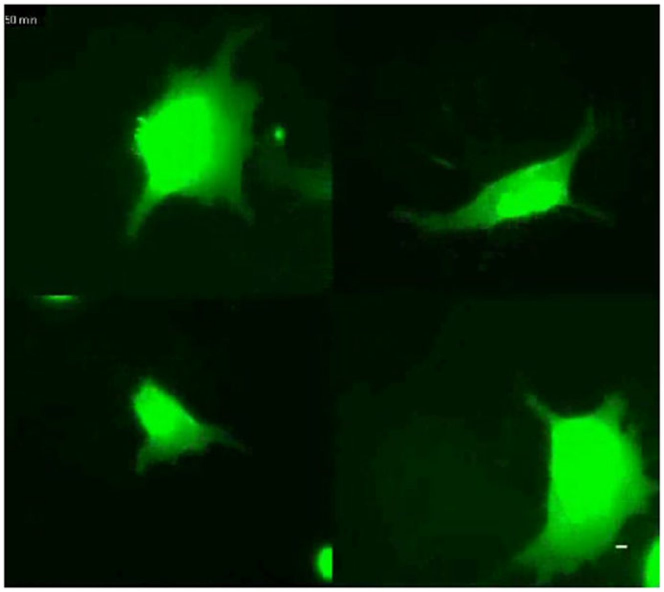

Movie 10. Observation of cells for 1 hr in 20× mag, stating from approx. 30 min from attaching to 10 μg/ml fibronectin coated surface. TH1+2+3 transfected cells were imaged every 2 min in a Nikon Biostation IMQ microscope. Movie was played at 20 fps. Bar 10 μm.

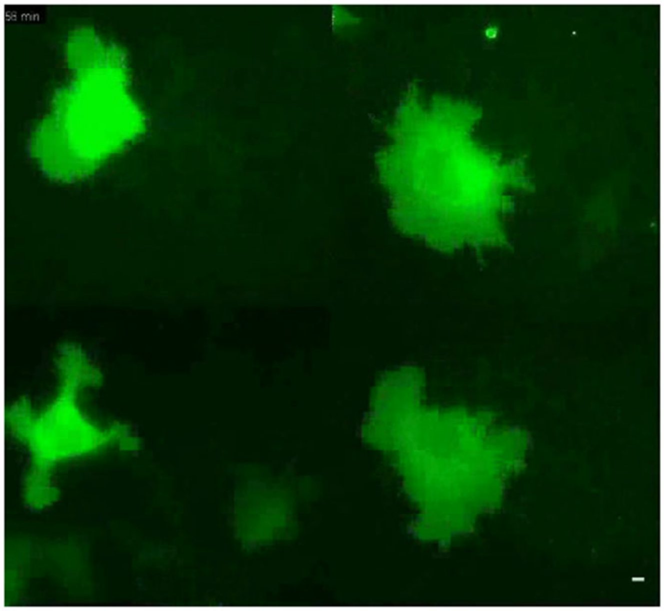

Movie 11. Observation of cells for 1 hr in 20× mag, stating from approx. 30 min from attaching to 10 μg/ml fibronectin coated surface. Myo1EΔTH3 transfected cells were imaged every 2 min in a Nikon Biostation IMQ microscope. Movie was played at 20 fps. Bar 10 μm.

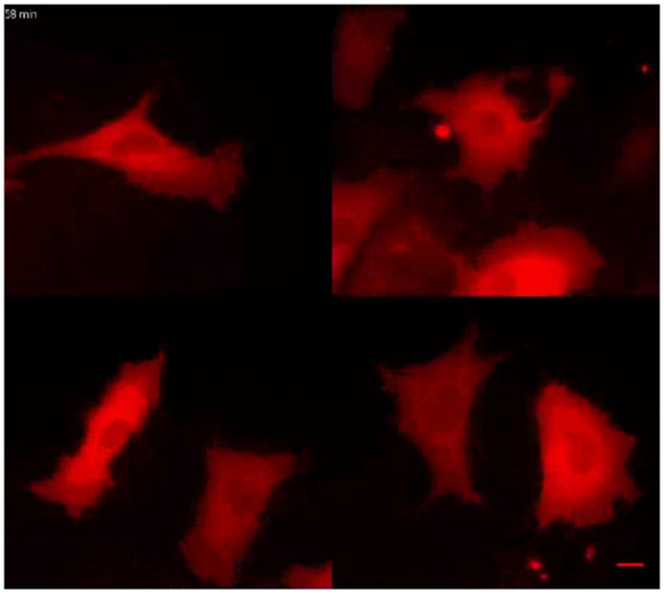

Movie 12. Observation of cells for 1 hr in 20× mag, stating from approx. 30 min from attaching to 10 μg/ml fibronectin coated surface. mApple-Myosin 1E transfected cells were imaged every 2 min in a Nikon Biostation IMQ microscope. Movie was played at 20 fps. Bar 10 μm.

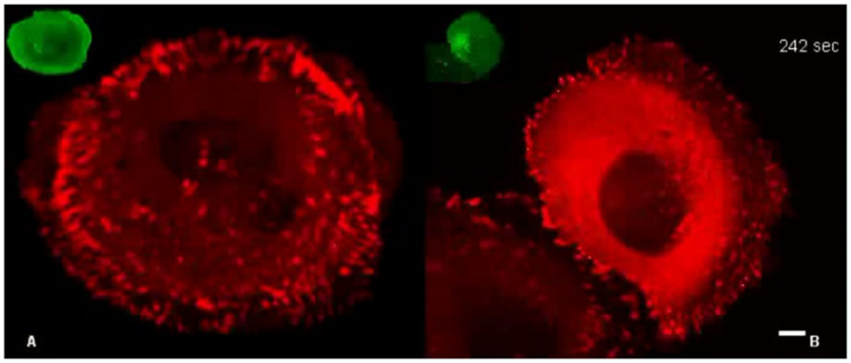

Movie 13. (A) RFP-Paxillin and co-expressing EGFP-Myosin 1EΔSH3 (inset), (B) RFP-Paxillin and co-expressing EGFP-(TH1+2+3) (inset), observed in Yokogawa confocal spinning disk microscope (based on a Nikon Ti system) every 2 sec by 60× lenses and played at 100 fps. Bar 5 μm.

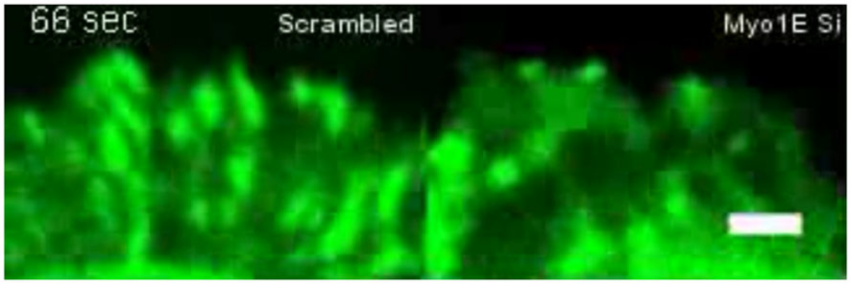

Movie 14. GFP-Paxillin patterns in contracting lamellipodia (imaged every 3 min, played at 20 fps), in scrambled siRNA transfected cells (left) and Myosin 1E transfected siRNA cells (right). Myosin 1E depletion made adhesions weak and paxillin staining round shaped. Bar 2 μm.

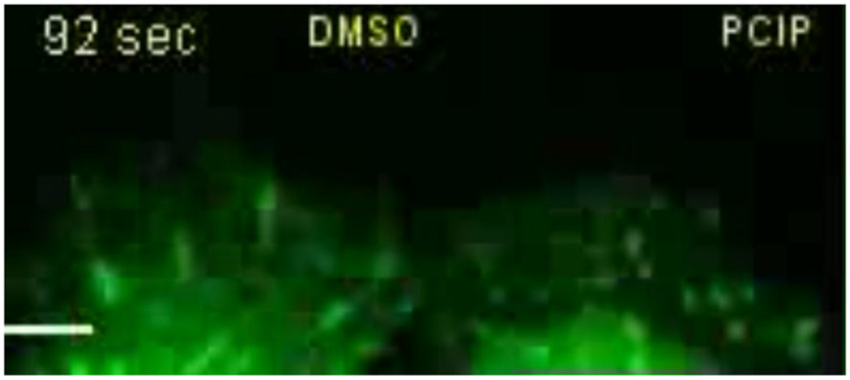

Movie 15. GFP-Paxillin patterns in contracting lamellipodia (imaged every 4 min, 20 fps), in Myo1 inhibitor PCIP treated cells (right) and untreated cells (left). Myosin 1E depletion made adhesions weak and paxillin staining round shaped. Bar 2 µm.

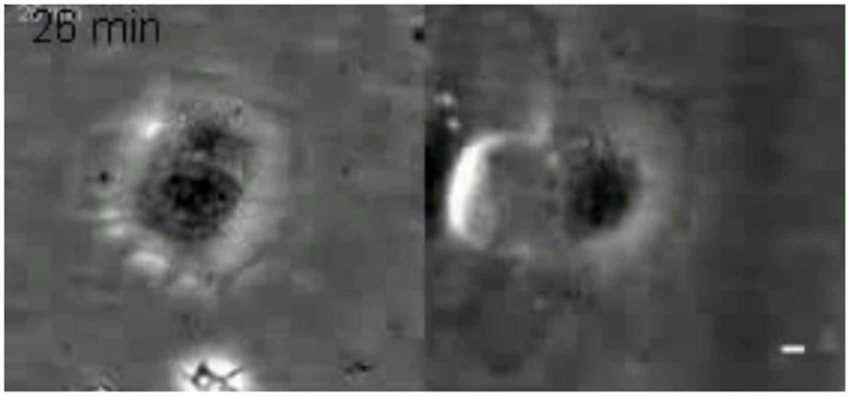

Movie 16. Two cells were allowed to spread and PCIP was added at 20 min and washed out at 130 min. Imaged every minute, played at 20 fps. Bar 5 µm.
